# Reference nodule transcriptomes for *Melilotus officinalis* and *Medicago sativa* cv. Algonquin

**DOI:** 10.1002/pld3.408

**Published:** 2022-06-08

**Authors:** Rui Huang, Wayne A. Snedden, George C. diCenzo

**Affiliations:** ^1^ Department of Biology Queen's University Kingston Ontario Canada

**Keywords:** legumes, NCR peptides, rhizobia, symbiotic nitrogen fixation, transcriptomics

## Abstract

Host/symbiont compatibility is a hallmark of the symbiotic nitrogen‐fixing interaction between rhizobia and legumes, mediated in part by plant‐produced nodule‐specific cysteine‐rich (NCR) peptides and the bacterial BacA membrane protein that can act as a NCR peptide transporter. In addition, the genetic and metabolic properties supporting symbiotic nitrogen fixation often differ between compatible partners, including those sharing a common partner, highlighting the need for multiple study systems. Here, we report high‐quality nodule transcriptome assemblies for *Medicago sativa* cv. Algonquin and *Melilotus officinalis* , two legumes able to form compatible symbioses with *Sinorhizobium meliloti* . The compressed *M. sativa* and *M. officinalis* assemblies consisted of 79,978 and 64,593 contigs, respectively, of which 33,341 and 28,278 were assigned putative annotations, respectively. As expected, the two transcriptomes showed broad similarity at a global level. We were particularly interested in the NCR peptide profiles of these plants, as these peptides drive bacterial differentiation during the symbiosis. A total of 412 and 308 NCR peptides were predicted from the *M. sativa* and *M. officinalis* transcriptomes, respectively, with approximately 9% of the transcriptome of both species consisting of *NCR* transcripts. Notably, transcripts encoding highly cationic NCR peptides (isoelectric point > 9.5), which are known to have antimicrobial properties, were ∼2‐fold more abundant in *M. sativa* than in *M. officinalis* , and ∼27‐fold more abundant when considering only NCR peptides in the six‐cysteine class. We hypothesize that the difference in abundance of highly cationic NCR peptides explains our previous observation that some rhizobial *bacA* alleles which can support symbiosis with *M. officinalis* are unable to support symbiosis with *M. sativa* .

## 1 INTRODUCTION

Leguminous plants are able to establish symbiotic relationships with a group of soil bacteria known as rhizobia. During the interaction, the rhizobia are located within a specialized organ known as a nodule where they fix atmospheric nitrogen into ammonia in exchange for reduced carbon from their host. Symbiosis is initiated following an exchange of chemical signals in the rhizosphere between compatible partners (Oldroyd, 2013): legumes secrete flavonoids that attract soil rhizobia and induce expression of rhizobial *nod* genes, leading to rhizobial production of chito‐oligosaccharide Nod factors that elicit the nodulation process by legumes. This process involves the curling of root hairs to trap rhizobia and the formation of infection threads within which rhizobia divide and move toward the root cortical layer (Gage, 2004). Rhizobia released from infection threads are internalized by nodule cells, where they develop into mature N_2_‐fixing bacteroids. In some legumes, such as those belonging to the Inverted Repeat Lacking Clade (IRLC), the rhizobia undergo an irreversible host‐induced process known as terminal differentiation that is largely driven by a unique class of legume proteins known as nodule‐specific cysteine‐rich (NCR) peptides (Van de Velde et al., 2010). Terminal differentiation involves cell enlargement, genome endoreplication, and increased membrane permeability and is thought to increase the efficiency of N_2_‐fixation (Haag & Mergaert, 2019; Lamouche et al., 2019; Mergaert et al., 2006).

Not all rhizobium/legume pairings are compatible (Pueppke & Broughton, 1999; Wilson, 1939). Partner compatibility is determined by numerous factors impacting both early and late stages of the symbiotic interaction (Walker et al., 2020). The flavonoids secreted by legumes vary, as does the ability of rhizobia to respond to different flavonoids (Kosslak et al., 1987; Maxwell et al., 1989; Pueppke et al., 1998; Recourt et al., 1991). Similarly, the Nod factor produced by rhizobia differ and legume hosts respond only to Nod factors with specific structures (DHaeze & Holsters, 2002). Moreover, legume infection depends on rhizobia producing particular host‐compatible exopolysaccharide molecules (Finan et al., 1985; Leigh et al., 1985), and variations in exopolysaccharide structure can impact specificity at the level of plant ecotype and bacterial strain (Simsek et al., 2007). In addition, some rhizobia secrete effector proteins that induce effector‐triggered immune responses in a cultivar‐specific manner, thereby influencing host range (Tsukui et al., 2013; Tsurumaru et al., 2015; Yang et al., 2010). Moreover, for IRLC legumes, an effective symbiotic interaction requires compatibility between the host‐produced NCR peptides and the rhizobial membrane protein BacA (diCenzo et al., 2017; Wang et al., 2017, 2018; Yang et al., 2017).

NCR peptides are a large class of legume‐specific proteins, with ∼600 members in *Medicago truncatula* (Young et al., 2011). These proteins display little conservation in amino acid composition but possess four or six cysteine residues at conserved positions (Mergaert et al., 2003). The length of mature NCR peptides varies from about 20 to 50 amino acids and includes two or three disulfide bridges (Maróti et al., 2015). NCR peptides can be classified as cationic (isoelectric point [pI] ≥ 8), neutral (6 ≤ pI < 8), or anionic (pI < 6) (Maróti et al., 2015). Highly cationic peptides (pI ≥ 9.0) display antimicrobial activity in vitro, likely through disrupting microbial membranes, thereby leading to permeabilization and cell lysis (Maróti et al., 2011; Tiricz et al., 2013). *In planta*, NCR peptides are required for rhizobium terminal differentiation and an effective symbiosis in IRLC legumes (Van de Velde et al., 2010). Deletion of individual *NCR* genes has shown that at least two of the ∼600 NCR peptides in *M. truncatula* are essential for N_2_‐fixation (Horváth et al., 2015; Kim et al., 2015); however, mutation of other *NCR* genes resulted in N_2_‐fixation in previously incompatible symbioses (Wang et al., 2017; Yang et al., 2017), demonstrating the role of NCR peptides in partner compatibility.

The ability of rhizobia to establish an effective symbiosis with IRLC legumes requires the membrane protein BacA (Glazebrook et al., 1993). BacA is a peptide transporter whose deletion results in multiple phenotypes including increased resistance to bleomycin, and gentamicin increased sensitivity to detergents, and altered membrane composition (Ferguson et al., 2004, 2002; Ichige & Walker, 1997; Marlow et al., 2009). In addition, *bacA* deletion mutants are both unable to import NCR peptides and show increased sensitivity to cationic NCR peptides (Barrière et al., 2017; Guefrachi et al., 2015; Haag et al., 2011). Moreover, rhizobium *bacA* mutants are unable to fix nitrogen in symbiosis with IRLC legumes; instead, the rhizobia are quickly killed in a NCR peptide‐dependent fashion upon release from the infection threads (Glazebrook et al., 1993; Haag et al., 2011). Intriguingly, BacA appears to be a host‐range determinant factor in IRLC legumes. For example, studies have shown that introduction of the *bacA* or *bacA*‐like genes of *Mesorhizobium loti* and *Bradyrhizobium* species into a *Sinorhizobium meliloti* *bacA* mutant is insufficient to allow N_2_‐fixation during interaction with IRLC legumes of the genus *Medicago* (Guefrachi et al., 2015; Maruya & Saeki, 2010). Similarly, we previously demonstrated that replacement of the *S. meliloti* *bacA* with the *bacA* alleles of *Sinorhizobium fredii* NGR234 or *Rhizobium leguminosarum* bv. *viciae* 3841 does not allow for N_2_‐fixation during symbiosis with *Medicago sativa* (alfalfa) but does support N_2_‐fixation on the IRLC legumes *Melilotus alba* (white sweet clover) and *Melilotus officinalis* (yellow sweet clover) (diCenzo et al., 2017 and Table S1).

In addition to the above‐noted comparison, several symbiotic differences have been observed when *S. meliloti* mutants interact with *Medicago* versus *Melilotus* plants (diCenzo et al., 2015; Geddes et al., 2021; Honma & Ausubel, 1987; Zamani et al., 2017), suggesting that *Melilotus* plants are a valuable secondary model system to study the symbiotic properties of *S. meliloti*. To further develop *M. officinalis* as a model species for studying symbiosis, here we report a reference nodule transcriptome for *M. officinalis*. We further compare the characteristics and the expression of *NCR* genes between *M. officinalis* and *M. sativa* to investigate whether the ability of certain *bacA* alleles to support symbiosis with *Melilotus* but not *Medicago* plants is correlated with differences in the NCR peptide profile of these genera.

## 2 MATERIALS AND METHODS

### 2.1 Plant materials and sample collection

*M. sativa* cv. Algonquin (alfalfa) and *M. officinalis* (yellow blossom sweet clover) seeds were purchased from Speare Seeds Limited (Harriston, Ontario, Canada). Seeds were surface sterilized with 95% ethanol for 5 min followed by 2.5% hypochlorite for 20 min and then soaked in sterile double‐distilled water (ddH_2_O) for 1 h. The sterilized seeds were plated on 1X water agar plates and incubated at room temperature in the dark for 2 days. Five germinated seeds were placed in autoclaved Leonard Assemblies consisting of two Magenta Jars with a cotton wick extending from the top jar (containing vermiculite mixed with silica sand [1:1 *w/w*]) into the bottom jar (containing 250 ml of Jensen's media; Jensen, 1942) and then incubated in a Conviron growth chamber for two nights. Wildtype *S. meliloti* strain Rm2011 was grown overnight at 30°C in LBmc broth (10 g L^−1^ tryptone, 5 g L^−1^ yeast extract, 5 g L^−1^ NaCl, 2.5 mM CaCl_2_, and 2.5 mM MgCl_2_), washed with 0.85% NaCl, and diluted to a density of ∼1 × 10^7^ CFU ml^−1^ in sterile ddH_2_O. Ten milliliters of cell suspension was then added to each Leonard Assembly. Plants were grown in a Conviron growth chamber with a day (18 h, 21°C, light intensity of 300 μmol m^−2^ s^−1^) and night (6 h, 17°C) cycle. Root nodules were collected 4 weeks postinoculation and immediately flash frozen with liquid N_2_ and stored at −80°C until use. All nodules collected from plants grown in the same Leonard Assembly were stored in a single tube and treated as one replicate. The shoots from each pot were dried at 60°C for 2 weeks prior to measuring shoot dry weight (Table S2).

### 2.2 RNA extraction and sequencing

Total RNA from three replicates of frozen *M. sativa* and *M. officinalis* nodule tissue was extracted using Direct‐zol RNA miniprep kits (ZYMO Research) according to the manufacturer's protocol. Total RNA samples were treated with DNase I (New England Biolabs) to degrade any contaminating DNA according to the manufacturer's protocol and the RNA again purified using Direct‐zol RNA miniprep kits. Total RNA samples were run on a MOPS‐formaldehyde agarose gel (119 ml MOPS buffer [200 mM MOPS, 80 mM sodium acetate, 10 mM EDTA, pH 7.0, in DEPC‐treated ddH_2_O], 6 ml formaldehyde, 1.25 g agarose) to check the integrity of the RNA (Figure S1) and subsequently verified using an Agilent Bioanalyzer chip.

Library preparation and Illumina sequencing were performed at The Centre for Applied Genomics at The Hospital for Sick Children (Toronto, Ontairo, Canada). Prior to library preparation, the quality of total RNA samples was checked on an Agilent Bioanalyzer 2100 RNA chip following Agilent Technologies' recommendation. RNA concentration was measured using Qubit RNA HS Assays on a Qubit fluorometer (ThermoFisher). RNA library preparation was performed following the NEBNext® Ultra™ II Directional RNA Library Prep Kit for Illumina® and the NEBNext Poly(A) mRNA Magnetic Isolation Module protocol. Briefly, 800 ng of total RNA was used as the input material and enriched for poly‐A mRNA using magnetic oligo d(T)25 beads, fragmented into the 200–300‐bases range for 10 min at 94°C and converted to double stranded cDNA. cDNA proceeded to library prep with dual‐index Illumina adapters added using PCR for seven cycles. One microliter of the final RNA libraries was loaded on a Bioanalyzer 2100 DNA High Sensitivity chip (Agilent Technologies) to check for size and quantified by qPCR using Kapa Library Quantification Illumina/ABI Prism Kit protocol (KAPA Biosystems). Validated libraries were pooled in equimolar quantities and paired‐end sequenced on the Illumina HiSeq 2500 High Throughput flowcell following Illumina's recommended protocol to generate paired‐end reads of 125‐bases in length.

### 2.3 Sequencing read trimming

Preprocessing of the raw reads was performed to ensure only high‐quality data were used for de novo transcriptome assembly and differential expression analysis. Read quality was initially evaluated using FastQC version 0.11.9 (Andrews et al., 2015), following which errors in raw reads were identified and corrected by the k‐mer‐based method of Rcorrector version 1.0.4 (Song & Florea, 2015). The FilterUncorrectablePEfastq.py script (github.com/harvardinformatics/TranscriptomeAssemblyTools/) was used to remove any read pairs where at least one read had an unfixable error identified by Rcorrector. Adaptors sequences, short reads (< 25 bp), and low‐quality reads (Q score < 20) were removed using Trim Galore version 0.6.6 (bioinformatics.babraham.ac.uk/projects/trim_galore/), which is a wrapper calling cutadapt version 3.2 (Martin, 2011) and FastQC. The processed reads were further trimmed by Trimmomatic version 0.4.0 (Bolger et al., 2014) included in the Trinity software distribution with the following parameters: *SLIDINGWINDOW:5:20 LEADING:3 TRAILING:3 MINLEN:25*. The quality and presence of adaptors in the preprocessed reads were then examined using FastQC. Between 38 and 94 million paired‐end reads remained per sample following preprocessing (Table S3), with a total of 174,707,055 and 119,333,821 paired‐end reads (∼43.7 and ∼29.8 Gb, respectively) remaining for *M. sativa* and *M. officinalis*, respectively (Table S4).

### 2.4 Transcriptome de novo assembly and quality control

The nodule transcriptomes of *M. sativa* and *M. officinalis* were de novo assembled following the same procedure. First, the preprocessed reads from the triplicate samples were simultaneously provided to Trinity version 2.9.0 for assembly without genome guidance (Grabherr et al., 2011). Then, the assembled contigs were clustered into gene‐level clusters using SuperTranscripts (Davidson et al., 2017). Gene isoforms were identified by Corset version 1.09 with the log likelihood ratio threshold set to very high (Davidson & Oshlack, 2014). Based on the Corset clusters, Lace version 1.14.1 was used to merge the gene isoforms into single long supertranscripts meant to provide a gene‐like view of the transcriptome (Davidson et al., 2017).

Multiple methods were used to examine the quality of the Trinity and SuperTranscript assemblies. First, the alignment rates of the preprocessed reads to the assemblies were inspected using STAR version 2.7.8a with the two‐pass mode that is more sensitive to alternative splicing (Dobin et al., 2013). Second, assembly statistics such as N50 and number of contigs were calculated using the seqstats software (github.com/clwgg/seqstats). Third, the completeness of the assemblies was evaluated using BUSCO version 5.1.2, run separately using the OrthoDB v10 ‘Fabales’ and ‘Viridiplantae’ reference databases (Seppey et al., 2019). The assemblies were also compared with the *S. meliloti* Rm2011 genome (Sallet et al., 2013) using BLASTn version 2.5.0 + (Camacho et al., 2009), which confirmed the absence of contaminating *S. meliloti* transcripts in the assemblies. Finally, the *M. sativa* de novo assembly was aligned to a publicly available genome of *M. sativa* cultivar XinJiangDaYe (Chen et al., 2020) with MUMmer version 4.0+, and 87.3% of transcripts were sucessfully aligned to the genome.

### 2.5 Transcriptome annotation

Coding regions within the supertranscripts were predicted by TransDecoder version 5.5.0 (github.com/TransDecoder/TransDecoder), using the results of BLASTp searches (E‐value cutoff of 1e‐5) against the Uniport database as ORF retention criteria (2021 January release) (The UniProt Consortium, 2021). The functional annotation of the predicted coding sequences then proceeded via three steps. First, BLAST bidirectional best hits between the *M. truncatula* A17 proteome (assembly release r5.0 1.7) (Pecrix et al., 2018) and the longest predicted protein isoform of each contig of our transcriptome assemblies were identified using BLASTp (E‐value cutoff of 1e‐5, culling limit 1). For all bidirectional best hits, the annotations from *M. truncatula* A17 were transferred to the corresponding contigs of the *M. sativa* or *M. officinalis* transcriptome. Second, all predicted protein isoforms of each contig in each transcriptome assembly were annotated using eggNOG‐mapper version 2.1.0 with DIAMOND version 2.0.4 and the Viridiplantae dataset (E‐value cutoff of 1e‐3) (Buchfink et al., 2015; Huerta‐Cepas et al., 2019). Third, for each contig not annotated by BLAST or eggNOG‐mapper, the hmmsearch function of HMMER version 3.3.2 was used to search all predicted protein isoforms against the complete set of hidden Markov models (HMMs) from the Pfam version 34.0 database and separately against the TIGRFAM version 15.0 HMM database (E‐value cutoff of 1e‐5) (Eddy, 2011; Haft et al., 2012; Mistry et al., 2021), and results were filtered to remove annotations with a Bit‐score < 50. For repetitive annotations from isoforms of a gene, only the consensus annotations were retained. For contigs successfully annotated by more than one of the annotation methods, results from the bidirectional BLAST took priority, followed by the results of eggNOG‐mapper, then the Pfam searches, and finally the TIGRFAM searches.

### 2.6 NCR peptide identification

Considering the high degree of sequence diversity of NCR peptide sequences, the functional annotation methods described above were not sufficiently sensitive to discover genes encoding NCR peptides in the assemblies. Therefore, the SPADA version 1.0 pipeline was used to identify NCR peptides (Zhou et al., 2013). SPADA is specialized to predict cysteine‐rich peptides in plant genomes and is distributed with a *M. truncatula* prediction model. Cysteine‐rich peptides in the *M. sativa* and *M. officinalis* assemblies were predicted using the SPADA pipeline with the following software: HMMER version 3.0, Augustus version 2.6, GeneWise version 2.2.0, GeneMark.hmm eukaryotic version 3.54, GlimmerHMM version 3.0.1, and GeneID version 1.1 (Birney et al., 2004; Blanco et al., 2007; Lukashin & Borodovsky, 1998; Stanke et al., 2006). The putative NCR peptide sequences were filtered to remove those without a signal peptide and then further filtered based on the E‐value (cutoff of 1e‐5) and hmm score (cutoff of 50). Filtered sequences were then verified via hmmscan searches against the Pfam database, and they were aligned using Clustal Omega version 1.2.4 (Sievers et al., 2011) to ensure the presence of the signature cysteine motif and N terminal signal peptide that are present in bona fide NCR peptides.

### 2.7 NCR peptides classification and clustering

To predict the lengths of mature NCR peptides, signalP version 4.1g with the notm network was used to predicted cleavage sites and extract mature NCR peptides (Petersen et al., 2011), and the number of cysteine residues in each motif were counted. The pI values of the NCR peptides were predicted using the pIR R package, and the value for each peptide was calculated based on the mean values from all prediction methods excluding the highest and lowest values (Audain et al., 2016). The *M. sativa*, *M. officinalis*, and *M. truncatula* NCR peptides were clustered using CD‐HIT‐2D with an identity threshold of 0.7 (Li & Godzik, 2006).

### 2.8 Gene‐expression level estimation and differential expression analysis

Gene‐expression levels were estimated individually for each library based on transcript abundance estimation using salmon version 0.12.0 in mapping‐based mode (library type automatic, validate Mapping) (Patro et al., 2017) and the reference transcriptomes produced as described above. R package deseq2 version 1.32.0 (Love et al., 2014) was used to perform differential expression analysis between *M. sativa* and *M. officinalis*, using the raw counts for each replicate from salmon, the length of each gene in each species as an additional parameter during normalization, and limiting the analysis to one‐to‐one orthologs identified by OrthoFinder version 2.5.2 (Emms & Kelly, 2019). OrthoFinder was run with default settings using the total predicted *M. sativa* and *M. officinalis* proteomes including all isoforms, following which orthologs were reduced to one per supertransript.

### 2.9 Gene ontology term analysis

The Gene Ontology (GO) terms for *M. sativa* and *M. officinalis* were obtained from the *M. truncatula* A17 proteome (assembly release r5.0 1.7) and annotations from eggNOG‐mapper. For transcripts annotated with GO terms from both sources, the consensus GO term annotations were retained. Then, the GO terms were reduced based on the Generic GO subset (download 10 August 2021).

### 2.10 Software information

All analyses were performed in an Ubuntu 20.04.2 LTS (Linux 5.8.0‐48‐generic) operation system or on the Compute Canada Graham cluster. Custom scripts were written in Python version 3.8.5, and bash. R version 3.6.3 was used during data analysis.

## 3 RESULTS AND DISCUSSION

### 3.1 Reference nodule transcriptomes for *M. officinalis* and *M. sativa* cv. Algonquin

To establish reference nodule transcriptomes of *M. sativa* cv. Algonquin and *M. officinalis* during symbiosis with *S. meliloti* Rm2011, the poly‐A enriched RNA from triplicate samples was sequenced using Illumina technology (2 × 125 bp paired‐end reads), generating ∼50 Gb (∼202 million paired‐end reads) and ∼35 Gb (∼139 million paired‐end reads) of data for *M. sativa* and *M. officinalis*, respectively (see Table S4 for sequencing statistics). The use of a poly‐A enrichment step meant that only plant transcripts were represented in the sequencing output. De novo assembly of the *M. sativa* sequencing data resulted in 253,871 contigs, while 192,165 de novo assembled contigs were produced for *M. officinalis*. Contigs expected to represent splice variants of a single gene were merged into so‐called “supertranscripts” using the SuperTranscripts program, resulting in compressed assemblies of 79,978 and 64,593 contigs for *M. sativa* and *M. officinalis*, respectively (Table 1). Transcriptomes were annotated as described in the Materials and Methods, resulting in putative annotations for 33,431 *M. sativa* contigs and 28,278 *M. officinalis* contigs (Datasets S1 and S2). Of these, ∼52% (*M. sativa*) and ∼58% (*M. officinalis*) are high confidence annotations as they were transferred from the *M. truncatula* whole genome annotation following identification of putative orthologs using a BLAST bidirectional best hit approach (Table S5). Considering that previous studies have predicted the presence of ∼23,000 long noncoding RNAs (lncRNAs) in *M. truncatula* (Wang et al., 2015) and ∼47,000 lncRNAs in the legume *Pisum sativum* (pea) (Kerr et al., 2017), we hypothesize that the majority of the unannotated *M. sativa* and *M. officinalis* transcripts reflect lncRNAs.

**TABLE 1:** Summary statistics from the de novo trinity and compressed (SuperTranscripts) nodule transcriptome assemblies

	*Medicago sativa*		*Melilotus officinalis*	
	Trinity assembly	SuperTranscripts	Trinity assembly	SuperTranscripts
Total number of contigs	253,871	79,978	192,165	64,593
Total number of base pairs (bp)	239,421,306	96,211,060	191,200,637	81,371,534
Average contig length (bp)	943	1,203	995	1,260
Median contig length (bp)	618	734	646	780
Contig N50 (bp)	1,466	1,912	1,584	1968
Minimum contig length (bp)	176	193	183	198
Maximum contig length (bp)	12,655	29,784	14,584	23,941
Overall alignment rate (%)	98.99	92.14	99.15	93.76

**FIGURE 1:**
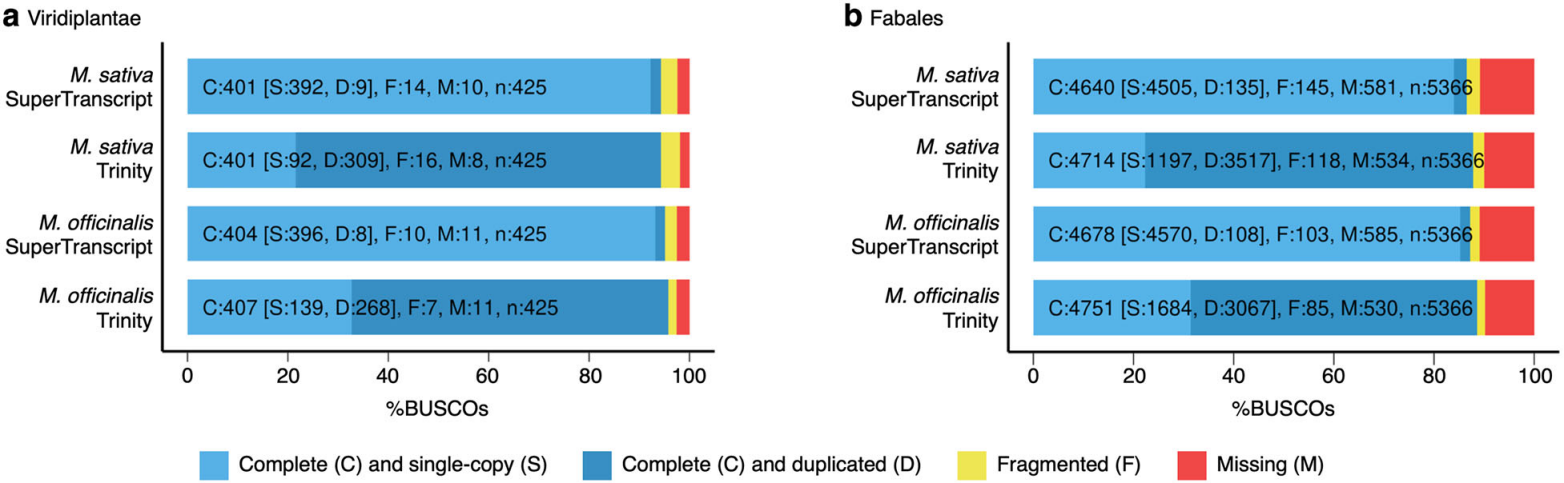
Estimates of nodule transcriptome completeness. Completeness of the *Medicago* *sativa* and *Melilotus officinalis* nodule transcriptome assemblies was assessed using BUSCO with the (a) Viridiplantae and (b) Fabales single‐copy marker gene datasets. The fraction of BUSCO genes identified as complete and single‐copy (light blue), complete but duplicated (dark blue), fragmented (yellow), and missing (red) is shown

All of the examined assembly summary statistics (mean and median contig length, contig N50) were improved in the compressed assemblies compared with the original de novo assemblies, indicating that the compressed assemblies are of higher structural quality (Table 1). The *M. sativa* transcriptome summary statistics, such as N50 and and transcript length, are consistent with those reported for other *M. sativa* de novo transcriptome assemblies, although the number of transcripts varies likely due to each study examining different tissues (Arshad et al., 2018; Zhang et al., 2015). In addition, the assemblies appear to be robust; greater than 90% of the filtered reads used for transcriptome assembly could be mapped to the corresponding assemblies by STAR (Table 1). Moreover, >92% and >83% of the Viridiplantae and Fabales BUSCO marker genes, respectively, were identified as complete and single‐copy in the *M. sativa* and *M. officinalis* compressed assemblies (Figure 1). The structural quality (e.g., high average and median contig length and N50) and BUSCO benchmark scores described here are in line with those reported for other plant de novo transcriptome assemblies (Al‐Qurainy et al., 2019; Malovichko et al., 2020; Weisberg et al., 2017). Taken together, these results indicate that our *M. sativa* cv. Algonquin and *M. officinalis* reference nodule transcriptomes are reliable and of high quality.

### 3.2 Comparative transcriptome analysis between *M. sativa* and *M. officinalis*

**FIGURE 2:**
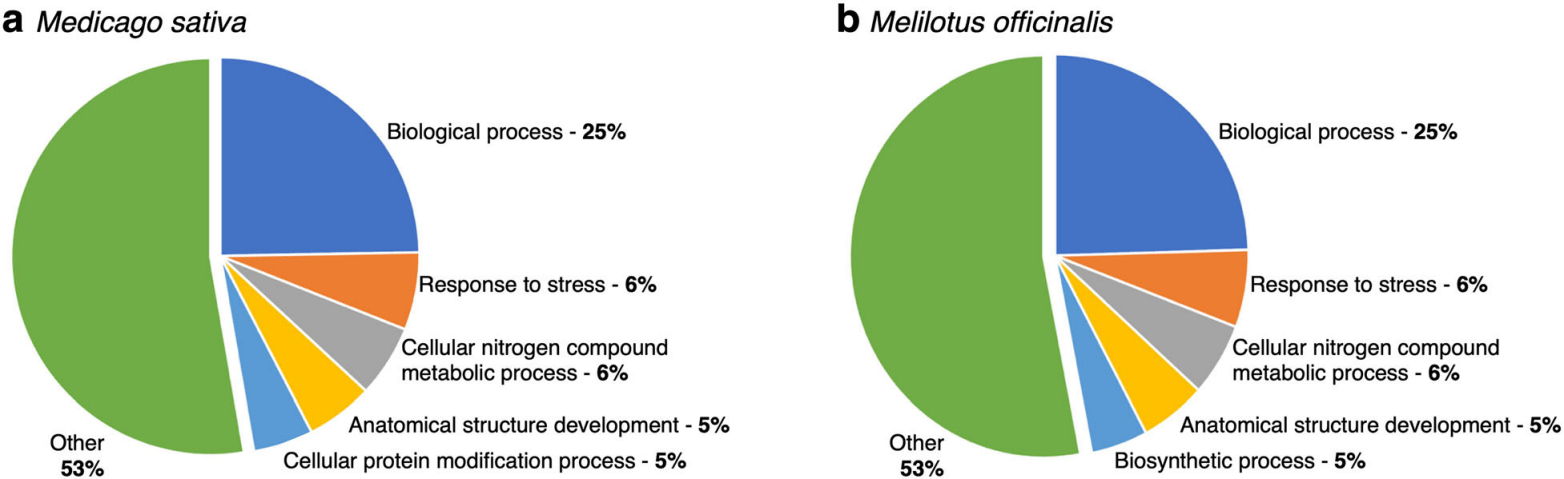
Summary of the slim GO biological processes annotations for the nodule transcriptomes. Transcripts were annotated with slim GO terms, and the annotations for the biological processes were summarized as pie charts for (a) *Medicago* *sativa* and (b) *Melilotus officinalis*

As an initial examination of the *M. sativa* and *M. officinalis* transcriptomes, the annotated functions of the proteins predicted to be encoded by the supertranscripts were summarized using the Generic GO term subset (Figure 2 and Datasets S3 and S4). Approximately 18,363 (26.1%) of the *M. sativa* supertranscripts and 16,674 (28.6%) of the *M. officinalis* supertranscripts were annotated with GO terms. No significant difference in the GO term profiles of the two species was observed, with the five most frequently annotated biological process GO terms being GO:0008150 (biological process), GO:0006950 (response to stress), GO:0006464 (cellular protein modification process), GO:003464 (cellular nitrogen compound metabolic process), and GO:0048856 (anatomical structure development). At this broad scale, the GO term data suggest that there is substantial similarity in the nodule transcriptomes of *M. sativa* and *M. officinalis*.

We next examined the predicted functions of the proteins encoded by the 50 most abundant transcripts in both species (Tables 2 and 3). Not surprisingly, these transcripts were enriched in those predicted to encode nodulins and leghaemoglobin‐like proteins. Nodulins refer to diverse proteins expressed specifically in nodule tissue, which play various structural or metabolic roles during symbiotic nitrogen fixation. Among the nodulins are the leghemoglobin proteins that account for up to 40% of the total soluble protein in legume nodules (Nash & Schulman, 1976). Leghemoglobins play an important role in maintaining the low free‐oxygen concentration required to protect the oxygen‐sensitive nitrogenase enzyme (Ott et al., 2005).

**TABLE 2:** The 50 most highly abundant transcripts in the *Medicago sativa* nodule transcriptome, with the average expression level in transcripts per million (TPM) and the functional annotation

Gene ID	TPM	Functional prediction
Cluster‐2.43518	30,662	Hypothetical protein (hypothetical leghaemoglobin)
Cluster‐2.26078	17,769	Putative albumin I
Cluster‐2.23447	12,515	Hypothetical protein (hypothetical leghaemoglobin)
Cluster‐2.23176	8,065	Nodulin‐25
Cluster‐2.22272	7,992	Putative late nodulin
Cluster‐2.23033	6,778	None
Cluster‐2.21873	5,919	Hypothetical protein
Cluster‐2.22935	5,253	Belongs to the globin family
Cluster‐2.33070	5,232	Putative ribonuclease H‐like domain‐containing protein
Cluster‐2.24197	4,245	Component of the replication protein A complex (RPA)
Cluster‐2.22983	4,196	Belongs to the globin family
Cluster‐2.24546	3,886	Predicted NCR peptide (crp1450_Cluster‐2.24546_0M_1)
Cluster‐2.19511	3,344	None
Cluster‐2.26207	3,296	Extensin‐like_protein_repeat
Cluster‐2.49512	3,173	Putative blue (type 1) copper binding protein
Cluster‐2.21810	3,050	Predicted NCR peptide (crp1160_Cluster‐2.21810_0M_1)
Cluster‐2.22936	3,014	Belongs to the globin family
Cluster‐2.29430	2,789	Putative late nodulin
Cluster‐2.22881	2,788	Predicted NCR peptide (crp1430_Cluster‐2.22881_0M_1)
Cluster‐2.22836	2,509	None
Cluster‐2.23729	2,271	Hypothetical protein
Cluster‐2.22458	2,245	Belongs to the globin family
Cluster‐2.24829	2,227	Hypothetical protein
Cluster‐2.25168	2,209	None
Cluster‐2.24278	2,093	Nodule‐specific_GRP_repeat
Cluster‐2.23928	2,038	Late_nodulin_protein
Cluster‐2.22042	2,029	None
Cluster‐2.23245	2,019	Putative translationally controlled tumor protein
Cluster‐2.25794	2,018	Putative protein‐synthesizing GTPase
Cluster‐2.31376	1,957	Predicted NCR peptide (crp1190_Cluster‐2.31376_0M_1)
Cluster‐2.21809	1,939	Predicted NCR peptide (crp1160_Cluster‐2.21809_0M_1)
Cluster‐2.28083	1,854	Predicted NCR peptide (crp1210_Cluster‐2.28083_0M_1)
Cluster‐2.23524	1,844	Early nodulin‐16
Cluster‐2.34649	1,839	Putative late nodulin
Cluster‐2.16993	1,808	Hypothetical protein
Cluster‐2.21813	1,731	None
Cluster‐2.22457	1,705	Belongs to the globin family
Cluster‐2.28283	1,674	None
Cluster‐2.26205	1,621	Predicted NCR peptide (crp1240_Cluster‐2.26205_0M_1)
Cluster‐2.23310	1,607	Asparagine synthetase
Cluster‐2.21870	1,596	Nodule‐specific_GRP_repeat
Cluster‐2.19604	1,583	Predicted NCR peptide (crp1420_Cluster‐2.19604_0M_1)
Cluster‐2.18485	1,509	Hypothetical protein
Cluster‐2.26876	1,458	Predicted NCR peptide (crp1410_Cluster‐2.26876_0M_1)
Cluster‐2.21536	1,441	Ubiquitin_family
Cluster‐2.22785	1,408	None
Cluster‐2.23374	1,385	Predicted NCR peptide (crp1420_Cluster‐2.23374_0M_1)
Cluster‐2.28787	1,311	Putative late nodulin
Cluster‐2.22402	1,292	Predicted NCR peptide (crp1520_Cluster‐2.22402_0M_1)
Cluster‐2.30081	1,289	Late_nodulin_protein

**TABLE 3:** The 50 most highly abundant transcripts in the *Melilotus* *officinalis* nodule transcriptome, with the average expression level in transcripts per million (TPM) and the functional annotation

Gene ID	TPM	Functional prediction
Cluster‐3554.18801	38,953	Belongs to the globin family
Cluster‐3554.18778	14,091	Belongs to the globin family
Cluster‐3554.16063	10,412	Late_nodulin_protein
Cluster‐3554.15387	7,885	Putative late nodulin
Cluster‐3554.16088	6,146	Putative late nodulin
Cluster‐3554.18892	6,104	None
Cluster‐3554.15771	5,813	Late_nodulin_protein
Cluster‐3554.18802	5,362	Belongs to the globin family
Cluster‐3554.18596	5,347	Predicted NCR peptide (crp1430_Cluster‐3554.18596_0M_1)
Cluster‐3554.15456	3,912	Putative translationally controlled tumor protein
Cluster‐3554.18808	3,864	Belongs to the globin family
Cluster‐3554.33215	3,302	Hypothetical protein
Cluster‐3554.18775	3,097	Putative late nodulin
Cluster‐3554.23000	2,990	Two predicted NCR peptide (crp1180_Cluster‐3554.23000_0M_1 and crp1180_Cluster‐3554.23000_0M_2)
Cluster‐3554.36681	2,877	Predicted NCR peptide (crp1500_Cluster‐3554.36681_0M_1)
Cluster‐3554.21555	2,868	Predicted NCR peptide (crp1430_Cluster‐3554.21555_0M_1)
Cluster‐3554.16074	2,809	Putative BURP domain‐containing protein
Cluster‐3554.29297	2,784	Hypothetical protein
Cluster‐3554.18196	2,723	None
Cluster‐3554.18256	2,571	Predicted NCR peptide (crp1440_Cluster‐3554.18256_0M_1)
Cluster‐3554.27063	2,522	None
Cluster‐3554.22577	2,449	Putative blue (type 1) copper binding protein
Cluster‐3554.15361	2,409	eEF1A
Cluster‐3554.21311	2,384	Belongs to the globin family
Cluster‐3554.18838	2,340	Late_nodulin_protein
Cluster‐3554.18700	2,296	Late_nodulin_protein
Cluster‐3554.11713	2,261	None
Cluster‐3554.18706	2,259	None
Cluster‐3554.23445	2,214	None
Cluster‐3554.17366	2,141	Predicted NCR peptide (crp1430_Cluster‐3554.17366_0M_1)
Cluster‐3554.23502	2,073	Hypothetical protein
Cluster‐3554.25153	2,009	Metallothionein‐like protein 2
Cluster‐3554.21451	1,953	Belongs to the globin family
Cluster‐3554.28600	1,931	Hypothetical protein
Cluster‐3554.22971	1,849	Hypothetical protein
Cluster‐3554.18877	1,812	Belongs to the glyceraldehyde‐3‐phosphate dehydrogenase family
Cluster‐3554.13172	1,732	Predicted NCR peptide (crp1440_Cluster‐3554.13172_0M_1)
Cluster‐3554.18195	1,704	Zinc_knuckle
Cluster‐3554.30751	1,653	Putative late nodulin
Cluster‐3554.21774	1,635	Late_nodulin_protein
Cluster‐3554.13784	1,629	Predicted NCR peptide (crp1420_Cluster‐3554.13784_0M_1)
Cluster‐3554.24033	1,611	Prolyl isomerase (PPIase)
Cluster‐3554.30294	1,556	Metallothionein‐like protein
Cluster‐3554.24764	1,552	None
Cluster‐3554.18368	1,552	Nucleoside diphosphate kinase 1
Cluster‐3554.25344	1,537	Metallothionein‐like protein 1
Cluster‐3554.23646	1,514	None
Cluster‐3554.9874	1,512	Belongs to the universal ribosomal protein uL13 family
Cluster‐3554.18476	1,503	Late_nodulin_protein
Cluster‐3554.31722	1,476	Putative late nodulin

**FIGURE 3:**
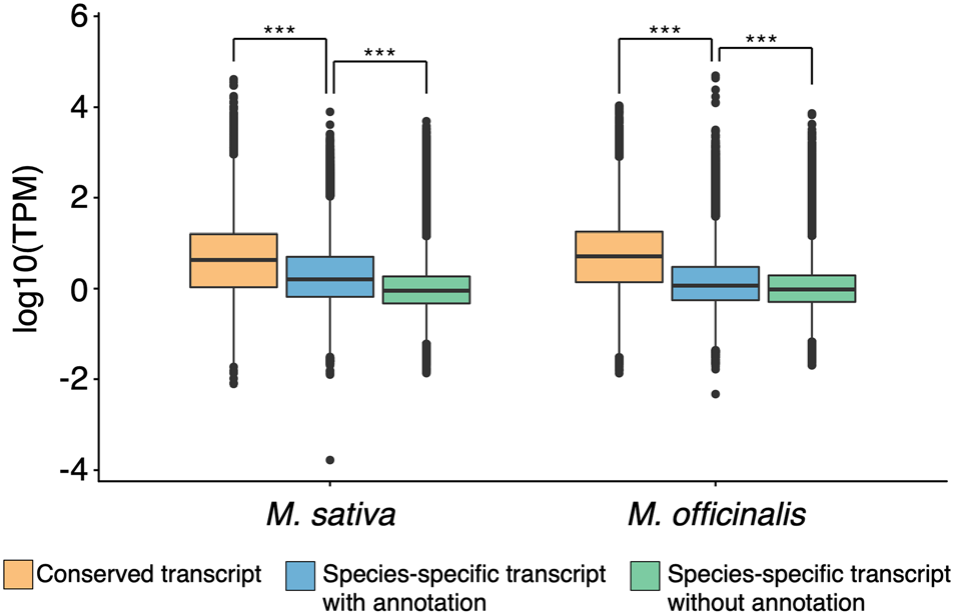
Transcript abundances for conserved and species‐specific transcripts. Box plots displaying the distribution of average transcript abundances from triplicate samples, shown separately for genes with orthologs in both *Medicago* *sativa* and *Melilotus officinalis* (orange), annotated transcripts found in only *M. sativa* or *M. officinalis* (blue), or transcripts that lack annotations and are found in only *M. sativa* or *M. officinalis* (green). Statistically significant differences between the distributions of a species are indicated with the asterisks (*p*‐value < 1e^−10^; pairwise Wilcox tests)

To facilitate further comparison of the *M. sativa* and *M. officinalis* transcriptomes, the proteins predicted to be encoded by the supertranscripts of both species were arranged into orthologous groups using OrthoFinder. A total of 20,237 orthologous groups, accounting for 26,304 *M. sativa* and 24,895 *M. officinalis* supertranscripts, were identified. Interestingly, the abundance of the conserved supertranscripts was significantly higher, on average, than that of the species‐specific transcripts (*p*‐value < 2.2e‐16; Figure 3). In both plant species, the majority of the most abundant, species‐specific annotated transcripts were also nodulins, globin family proteins that are likely species‐specific leghaemoglobin isoforms, and some housekeeping genes such as ribonuclease and ribosomal proteins. It is noteworthy that the most abundant *M. sativa* *‐*specific supertranscript is predicted to encode albumin I. Similarly, *M. officinalis* also has a highly expressed albumin I supertranscript. The albumin I peptide family is known to be highly expressed in legume seeds and play roles in seed protection (Rahioui et al., 2014). Expression of albumin I genes has also been observed in *M. truncatula* root nodules, with expression specific to uninfected cells in the nitrogen fixation zone (Limpens et al., 2013). These cells are thought to play essential roles in metabolite transport during symbiosis, and albumin I may have a role in protecting some of the nodule cells from rhizobium infection (Limpens et al., 2013). A phylogenetic analysis of *M. truncatula* nodulins and albumin I peptides indicated that the *M. truncaula* albumin I clustered with a subset of nodulins, reflecting a close evolutionary relationship between these proteins (Karaki et al., 2016).

**FIGURE 4:**
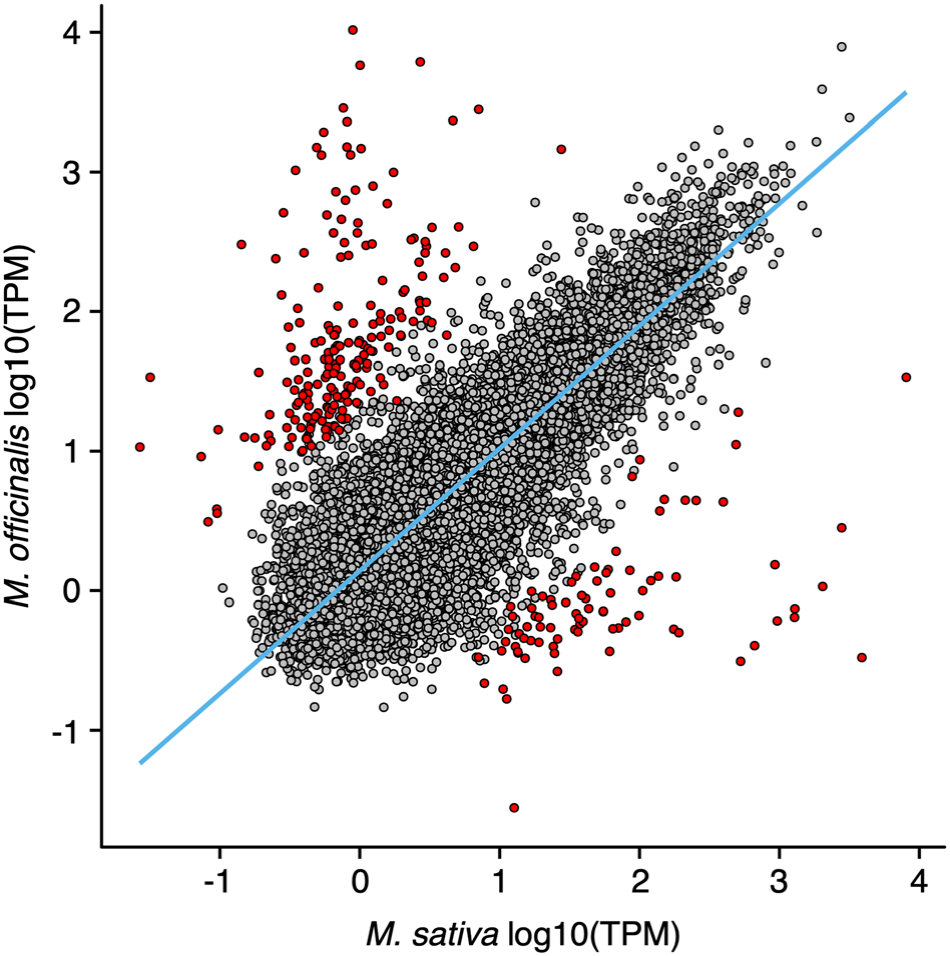
Correlation between transcript abundances of orthologous transcripts in *Medicago* *sativa* and *Melilotus officinalis* . Each datapoint represents the transcript abundance of single‐copy orthologous transcripts in *M. sativa* and *M. officinalis* . Red datapoints represent transcripts that are differentially abundant between the two species (|log_2_[fold change]| > 5, adjusted *p*‐value < .05); all other datapoints are in gray. The blue line represents the robust linear regression line, calculated with the rlm function of the MASS package in R

We next compared the abundances of supertranscripts conserved in both *M. sativa* and *M. officinalis*, limiting the analysis to the 15,287 one‐to‐one orthologs detected by OrthoFinder. Despite significant variation in the abundance of orthologous transcripts between *M. sativa* and *M. officinalis*—which may reflect limitations of interspecies transcriptome analysis—a clear correlation in the abundance of orthologous transcripts was detected (residual standard error = .517; Figure 4). Considering the limitations of interspecies differential expression analyses, we restricted our investigation to supertranscripts with absolute log_2_ fold changes > 5 and a *p*‐value < .05. Using these thresholds, we identified 290 differentially abundant transcripts, 86 of which were more abundant in *M. sativa*, and 204 of which were more abundant in *M. officinalis*. It should be noted, however, that only 159 of the differentially abundant transcripts were annotated with the same or similar function in both species, and we focus on these 159 transcripts in the following discussion.

Many of the differentially abundant conserved supertranscripts have annotated functions that suggest that the encoded proteins may impact symbiotic nitrogen fixation. These include 21 supertranscripts annotated as encoding nodulins, which include 16 that are more abundant in *M. officinalis* and five that are more abundant in *M. sativa*. In addition, 32 supertranscripts encoding proteins predicted to be associated with transcription and translation activity were differentially abundant, with 24 more abundant in *M. sativa* and eight more abundant in *M. officinalis*. We also observed that several supertranscripts encoding proteins predicted to be involved in cell wall synthesis or modification were differentially abundant, with six more highly abundant in *M. sativa* and one more highly abundant in *M. officinalis*. Other differentially abundant transcripts included those predicted to encode proteins involved in transport (17 transcripts), fatty acid biosynthesis (three transcripts), flavonoid biosynthesis (three transcripts), and aromatic compound biosynthesis (one transcript). Given that this analysis compares two plant species with differing growth rates (Table S2), we cannot rule out that some of these transcriptomic differences may also reflect variances in nodule maturity and/or host metabolic activity at the time of harvest.

### 3.3 NCR peptide diversity and expression profile

**FIGURE 5:**
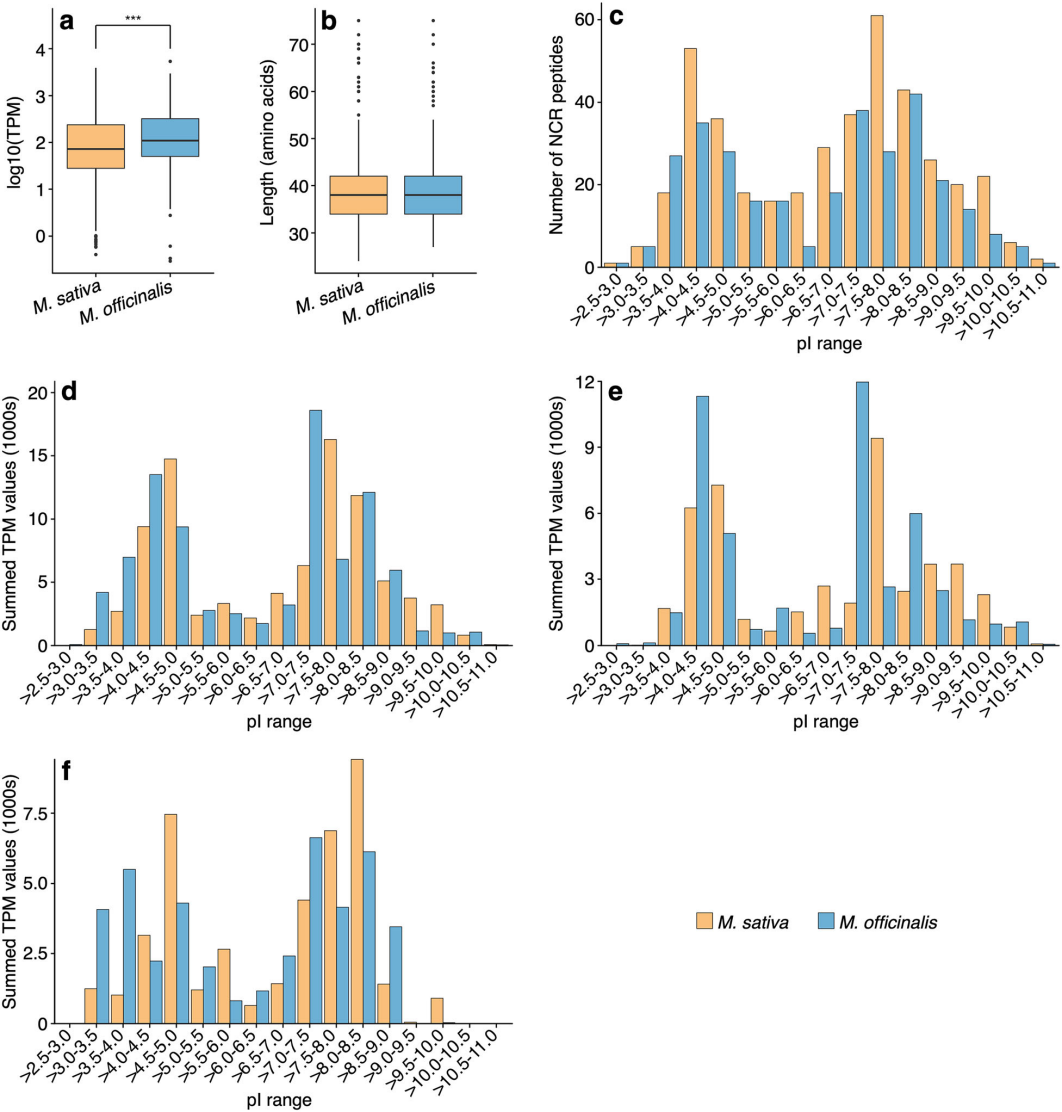
Nodule‐specific cysteine‐rich (NCR) peptide profiles of *Medicago sativa* and *Melilotus officinalis* . NCR peptides were predicted from the *M. sativa* (orange) and *M. officinalis* (blue) transcriptome assemblies, and the properties of the NCR peptides are shown in these graphs. (a) Box plots showing the distribution of the abundance (in transcripts per million, TPM) of *NCR* transcripts, based on triplicate samples. The difference in the distributions for the two species was statistically significant (*p*‐value < .001; pairwise Wilcox test). (b) Box plots showing the distribution of the amino acid lengths of mature NCR peptides. No statistically significant difference in the distributions for the two species was detected. (c, d) Histograms showing the distributions of the isoelectric points (pI) for the mature NCR peptides. Histograms are based either on the number of NCR peptides with a given pI value (C) or the total abundance of the transcripts encoding NCR peptides with a given pI value (D). (e, f) Histograms showing distributions of pI for 4‐cysteines (e) and 6‐cysteines (f) mature NCR peptides based on total abundance of the transcripts encoding NCR peptides with a given pI value

We previously observed that replacing the *bacA* allele of *S. meliloti* Rm2011 with the *bacA* alleles of the rhizobia *S. fredii* NGR234 or *R. leguminosarum* bv. *viciae* 3841 resulted in an inability to fix nitrogen with *M. sativa*, while the ability to fix nitrogen with *M. alba* and *M. officinalis* remained (diCenzo et al., 2017 and Table S1). We hypothesized that this was due to differences in the NCR peptide profiles of these species (diCenzo et al., 2017). To test this hypothesis, supertranscripts encoding NCR peptides were identified in the *M. sativa* and *M. officinalis* transcriptome assemblies using the SPADA pipeline (Zhou et al., 2013). A total of 412 and 308 supertranscripts encoding NCR peptides were identified in the *M. sativa* and *M. officinalis* transcriptomes, respectively, accounting for ∼0.5% of all supertranscripts in both assemblies (Datasets S5 and S6). The lower count of *NCR* transcripts in *M. officinalis* was offset by a higher median transcript abundance (58.6 transcripts per million [TPM] vs 99.1 TPM; *p* < .001; Figure 5a), resulting in *NCR* transcripts accounting for roughly 9% of the total nodule transcriptome in both species.

The number of NCR peptides predicted from the *M. sativa* transcriptome is similar to the 469 predicted previously based on a *M. sativa* genome sequence (Montiel et al., 2017). However, only 70% of the NCR peptides showed >70% identity, which could be due to the use of different cultivars and/or the method of NCR peptide prediction. Additionally, 63% of the *M. sativa* NCR peptides, but only 23% of *M. officinalis* NCR peptides, showed >70% sequence identity to NCR peptides from *M. truncatula* (Dataset S7). Notably, using a 70% identity threshold, only 40 NCR peptides were found in all three species, while two NCR peptides known to be essential for symbiosis in *M. truncatula* (NCR211 and NCR169) were not identified in *M. sativa* nor *M. officinalis* (Horváth et al., 2015; Kim et al., 2015). Overall, these results are consistent with rapid evolution of the *NCR* gene family in legumes and with the two *Medicago* species having NCR peptide profiles more similar to each other than to *M. officinalis*.

In both *M. sativa* and *M. officinalis*, NCR peptides had median lengths of 38 residues, with approximately half of the NCR peptides containing between 30 and 40 residues (Figure 5b). Additionally, there was a roughly even number of four‐ and six‐cysteine NCR peptides expressed in both plant species, with the four‐cysteine class of NCR peptides accounting for 51%–55% of the *NCR* transcripts both in terms of number of NCR peptides and expression of *NCR* transcripts as measured by TPM. The NCR peptides from both hosts also showed broadly similar distributions of pI values between approximately 3 to 11, with one peak around a pI of 4 and another around pI 8 (Figure 5c,d). The pI pattern of the NCR peptides we observed is reminiscent of that reported for other legume species that induce an elongated branched morphology in their microsymbiont, including *M. sativa* and *M. truncatula* (Montiel et al., 2017). Overall, at a global level, the property profiles of NCR peptides for *M. sativa* and *M. officinalis* were very similar, suggesting that the impact of different *bacA* alleles on symbiotic compatibility of *S. meliloti* with *M. sativa* is unlikely a consequence of global differences in the NCR peptide profiles of these plants and is more likely due to specific NCR peptides. Identifying which NCR peptides functionally correlate with symbiotic compatibility should be the focus of future studies.

Despite the general similarity in the NCR peptide profiles of *M. sativa* and *M. officinalis*, a key difference emerges when examining the abundance of NCR peptides with extreme pI values; transcripts encoding highly cationic NCR peptides were more abundant in *M. sativa*, while transcripts encoding highly anionic NCR peptides were more abundant in *M. officinalis* (Figure 5d). Previous work has shown that, in general, only cationic NCR peptides with a pI > 9.0 have antimicrobial activity (Lima et al., 2020), with anticandidal activity primarily limited to NCR peptides with a pI > 9.5 (Ördögh et al., 2014). Here, we observed that transcripts encoding highly cationic NCR peptides (pI > 9.0) were ∼2.4‐fold more abundant in *M. sativa* than *M. officinalis* (Figure 5d). Similarly, transcripts encoding NCR peptides with pI values > 9.5 were ∼1.9‐fold more abundant in *M. sativa* than *M. officinalis*. Notably, previous work indicated that 4.0% of *M. truncatula* *NCR* transcripts encode NCR peptides with pI values > 9.5, compared with only 1.8% in the *R. leguminosarum* bv. *viciae* symbiont *P. sativum* (Alves‐Carvalho et al., 2015; Montiel et al., 2017; Roux et al., 2014); this compares to 4.7% and 2.3% for *M. sativa* and *M. officinalis*, respectively (Figure 5c). Strikingly, when subdividing the NCR peptides with pI values > 9.5 into those with four or six cysteine residues, we observed that those with six cysteines were ∼27‐fold more abundant in *M. sativa* than *M. officinalis* (Figure 4e,f). Considering these results, we hypothesize that the ability of the *R. leguminosarum* *bacA* allele to support symbiosis with *M. officinalis* and *P. sativum*, but not *M. sativa*, is a consequence of the elevated abundance of highly cationic (pI > 9.5) NCR peptides in *Medicago* nodules. Consistent with this, two *M. sativa* NCR peptides were identified that had high sequence idenitity (83% and 73%) with *M. truncatula* NCR035, which is a cationic NCR peptide with antimicrobial activity against *S. meliloti* (Haag et al., 2011), whereas similar sequences were not found in *M. officinalis*. It may be that the BacA proteins of *S. fredii* and *R. leguminosarum* are less capable of transporting highly cationic NCR peptides, and consequently, strains with these BacA proteins may be more sensitive to the antimicrobial activities of these NCR peptides.

## 4 CONCLUSION

We report high‐quality nodule transcriptome assemblies for *M. sativa* cv. Algonquin and *M. officinalis* that we expect will serve as valuable resources for the legume research community. In particular, we expect that the availability of a nodule transcriptome for *M. officinalis* will help establish this plant as a secondary model system for studies of the symbiotic properties of *S. meliloti*.

We were particularly interested in using these transcriptomes to compare the properties of the NCR peptides encoded by both species. Despite predicting 33% more NCR peptides in *M. sativa* than *M. officinalis*, *NCR* transcripts accounted for roughly 9% of the transcriptome (based on TPM values) in both species. In general, the characteristics of the NCR peptides of *M. sativa* and *M. officinalis* were highly similar. However, transcripts encoding cationic NCR peptides with a pI > 9.5 were ∼2‐fold more abundant in *M. sativa* than in *M. officinalis* and 27‐fold more abundant when considering only six‐cysteine NCR peptides. These results are consistent with previous observations that transcripts encoding cationic NCR peptides with a pI > 9.5 account for ∼2‐fold more *NCR* transcripts in *M. truncatula* compared with *P. sativum*. Cationic, but not neutral or anionic, NCR peptides display antimicrobial activity through disrupting the integrity of microbial membranes (Mikuláss et al., 2016). It has been hypothesized that BacA provides protection against these NCR peptides by importing them into the cytoplasm and thus away from the membrane (Arnold et al., 2017; Nicoud et al., 2021). Considering that the BacA proteins of *S. fredii* and *R. leguminosarum* share less than 60% amino acid identity with the BacA protein of *S. meliloti*, it is reasonable to speculate that they have different substrate specificity and may be less capable of transporting cationic NCR peptides (diCenzo et al., 2017). If true, this could explain why the *bacA* alleles of *S. fredii* and *R. leguminosarum* can support symbiotic nitrogen fixation with *M. officinalis* but not *M. sativa*; the increased production of cationic NCR peptides in *M. sativa*, coupled with lower rates of import into the *S. meliloti* cytoplasm, could result in an accumulation of these peptides in the periplasm, resulting in a loss of viability and lack of nitrogen fixation (diCenzo et al., 2017). In future work, it will be interesting to test whether *S. meliloti* strains with different *bacA* alleles display differing sensitivities to these highly cationic NCR peptides or differences in their abilities to transport these peptides.

## CONFLICT OF INTEREST

The authors declare that they have no conflict of interest.

## Supporting information


**Dataset S1.** Annotation of the *Medicato sativa* nodule transcriptome.

**Dataset S2.** Annotation of the *Melilotus officinalis* nodule transcriptome.

**Dataset S3.** Summary of the GO slim analysis of the *Medicago sativa* nodule transcriptome assembly.

**Dataset S4.** Summary of the GO slim analysis of the *M. officinalis* nodule transcriptome assembly.

**Dataset S5.** NCR peptides predicted from the *M. sativa* nodule transcriptome assembly.

**Dataset S6.** NCR peptides predicted from the *M. officinalis* nodule transcriptome assembly.

**Dataset S7.** Summary of *M. sativa* and *M. officinalis* NCR peptides amino acid sequences comparsion with *Medicago truncatula* NCR peptides.

**Table S1.** Shoot dry weights of *M. sativa* and *M. officinalis* plants inoculated with *Sinorhizobium meliloti* strains carrying various *bacA* constructs.
**Table S2.** Shoot dry weights of *M. sativa* and *M. officinalis* plants inoculated with wildtype *S. meliloti* Rm2011 and used for the transcriptome analyses.
**Table S3.** Number of Illumina paired‐end reads remaining per library following preprocessing.
**Table S4.** Per species summary statistics from the preprocessing of the Illumina reads.
**Table S5.** Summary statistics from annotation of the compressed transcriptome assemblies.
**Figure S1. Integrity of the RNA samples used for RNA‐seq library preparation.** RNA samples were run on a MOPS‐formaldehyde agarose gel and imaged. The bands corresponding to the 28S rRNA, 18S rRNA, and 5.8S rRNA are indicated. The lack of smearing indicates that the purified RNA was of high quality and not degraded.

## Data Availability

All custom scripts to perform the analyses described in this study are available through GitHub (github.com/hyhy8181994/Nodule_transcriptome_script). Raw Illumina data are available through the Short Read Archive (SRR15724671, SRR15724670, SRR15724669, SRR15724668, SRR15724667, and SRR15724666) hosted by the National Center for Biotechnology Information (NCBI). The assembled transcriptomes are available through the Transcriptome Shotgun Assembly Sequence Database (GJLW00000000 and GJLK00000000) hosted by the NCBI.

## References

[pld3408-bib-0001] Al‐Qurainy, F. , Alshameri, A. , Gaafar, A.‐R. , Khan, S. , Nadeem, M. , Alameri, A. A. , Tarroum, M. , & Ashraf, M. (2019). Comprehensive stress‐based de novo transcriptome assembly and annotation of guar (*Cyamopsis tetragonoloba* (L.) Taub.): An important industrial and forage crop. International Journal of Genomics, 2019, 7295859. 10.1155/2019/7295859 31687376 PMC6800914

[pld3408-bib-0002] Alves‐Carvalho, S. , Aubert, G. , Carrère, S. , Cruaud, C. , Brochot, A. L. , Jacquin, F. , Klein, A. , Martin, C. , Boucherot, K. , Kreplak, J. , Da Silva, C. , Moreau, S. , Gamas, P. , Wincker, P. , Gouzy, J. , & Burstin, J. (2015). Full‐length de novo assembly of RNA‐seq data in pea (*Pisum sativum* L.) provides a gene expression atlas and gives insights into root nodulation in this species. The Plant Journal, 84, 1–19. 10.1111/tpj.12967 26296678

[pld3408-bib-0003] Andrews, S. , Krueger, F. , Seconds‐Pichon, A. , Biggins, F. , & Wingett, S. (2015). FastQC. A Quality Control Tool for High Throughput Sequence Data. Babraham Bioinformatics. Babraham Institute.

[pld3408-bib-0004] Arnold, M. F. F. , Shabab, M. , Penterman, J. , Boehme, K. L. , Griffitts, J. S. , & Walker, G. C. (2017). Genome‐wide sensitivity analysis of the microsymbiont *Sinorhizobium meliloti* to symbiotically important, defensin‐like host peptides. *m* Bio, 8(4), e01060–e01017. 10.1128/mBio.01060-17 PMC5539429 28765224

[pld3408-bib-0005] Arshad, M. , Gruber, M. Y. , & Hannoufa, A. (2018). Transcriptome analysis of microRNA156 overexpression alfalfa roots under drought stress. Scientific Reports, 8, 1–13. 10.1038/s41598-018-27088-8 29921939 PMC6008443

[pld3408-bib-0006] Audain, E. , Ramos, Y. , Hermjakob, H. , Flower, D. R. , & Perez‐Riverol, Y. (2016). Accurate estimation of isoelectric point of protein and peptide based on amino acid sequences. Bioinformatics, 32(6), 821–827. 10.1093/bioinformatics/btv674 26568629 PMC5939969

[pld3408-bib-0007] Barrière, Q. , Guefrachi, I. , Gully, D. , Lamouche, F. , Pierre, O. , Fardoux, J. , Chaintreuil, C. , Alunni, B. , Timchenko, T. , Giraud, E. , & Mergaert, P. (2017). Integrated roles of BclA and DD‐carboxypeptidase 1 in *Bradyrhizobium* differentiation within NCR‐producing and NCR‐lacking root nodules. Scientific Reports, 7, 1–13. 10.1038/s41598-017-08830-0 28831061 PMC5567381

[pld3408-bib-0008] Birney, E. , Clamp, M. , & Durbin, R. (2004). GeneWise and genomewise. Genome Research, 14(5), 988–995. 10.1101/gr.1865504 15123596 PMC479130

[pld3408-bib-0009] Blanco, E. , Parra, G. , & Guigó, R. (2007). Using geneid to identify genes. Current Protocols in Bioinformatics, 18, 3–4. 10.1002/0471250953.bi0403s18 18428791

[pld3408-bib-0010] Bolger, A. M. , Lohse, M. , & Usadel, B. (2014). Trimmomatic: A flexible trimmer for Illumina sequence data. Bioinformatics, 30(15), 2114–2120. 10.1093/bioinformatics/btu170 24695404 PMC4103590

[pld3408-bib-0011] Buchfink, B. , Xie, C. , & Huson, D. H. (2015). Fast and sensitive protein alignment using DIAMOND. Nature Methods, 12, 59–60. 10.1038/nmeth.3176 25402007

[pld3408-bib-0012] Camacho, C. , Coulouris, G. , Avagyan, V. , Ma, N. , Papadopoulos, J. , Bealer, K. , & Madden, T. L. (2009). BLAST+: Architecture and applications. BMC Bioinformatics, 10, 1–9. 10.1186/1471-2105-10-421 20003500 PMC2803857

[pld3408-bib-0013] Chen, H. , Zeng, Y. , Yang, Y. , Huang, L. , Tang, B. , Zhang, H. , Hao, F. , Liu, W. , Li, Y. , Liu, Y. , Zhang, X. , Zhang, R. , Zhang, Y. , Li, Y. , Wang, K. , He, H. , Wang, Z. , Fan, G. , Yang, H. , … Qiu, Q. (2020). Allele‐aware chromosome‐level genome assembly and efficient transgene‐free genome editing for the autotetraploid cultivated alfalfa. Nature Communications, 11, 2494. 10.1038/s41467-020-16338-x PMC7237683 32427850

[pld3408-bib-0015] Davidson, N. M. , Hawkins, A. D. K. , & Oshlack, A. (2017). SuperTranscripts: A data driven reference for analysis and visualisation of transcriptomes. Genome Biology, 18, 1–10. 10.1186/s13059-017-1284-1 28778180 PMC5543425

[pld3408-bib-0016] Davidson, N. M. , & Oshlack, A. (2014). Corset: Enabling differential gene expression analysis for de novo assembled transcriptomes. Genome Biology, 15(7), 1–14. 10.1186/s13059-014-0410-6 PMC4165373 25063469

[pld3408-bib-0017] DHaeze, W. , & Holsters, M. (2002). Nod factor structures, responses, and perception during initiation of nodule development. Glycobiology, 12(6), 79R–105R. 10.1093/glycob/12.6.79R 12107077

[pld3408-bib-0018] diCenzo, G. C. , Zamani, M. , Cowie, A. , & Finan, T. M. (2015). Proline auxotrophy in *Sinorhizobium meliloti* results in a plant‐specific symbiotic phenotype. Microbiology, 161(12), 2341–2351. 10.1099/mic.0.000182 26395514

[pld3408-bib-0019] diCenzo, G. C. , Zamani, M. , Ludwig, H. N. , & Finan, T. M. (2017). Heterologous complementation reveals a specialized activity for BacA in the *Medicago*–*Sinorhizobium meliloti* symbiosis. Molecular Plant‐Microbe Interactions, 30(4), 312–324. 10.1094/MPMI-02-17-0030-R 28398123

[pld3408-bib-0020] Dobin, A. , Davis, C. A. , Schlesinger, F. , Drenkow, J. , Zaleski, C. , Jha, S. , Batut, P. , Chaisson, M. , & Gingeras, T. R. (2013). STAR: Ultrafast universal RNA‐seq aligner. Bioinformatics, 29, 15–21. 10.1093/bioinformatics/bts635 23104886 PMC3530905

[pld3408-bib-0021] Eddy, S. R. (2011). Accelerated profile HMM searches. PLoS Computational Biology, 7(10), e1002195. 10.1371/journal.pcbi.1002195 22039361 PMC3197634

[pld3408-bib-0022] Emms, D. M. , & Kelly, S. (2019). OrthoFinder: Phylogenetic orthology inference for comparative genomics. Genome Biology, 20, 1–14. 10.1186/s13059-019-1832-y 31727128 PMC6857279

[pld3408-bib-0023] Ferguson, G. P. , Datta, A. , Baumgartner, J. , Roop, R. M. , Carlson, R. W. , & Walker, G. C. (2004). Similarity to peroxisomal‐membrane protein family reveals that *Sinorhizobium* and *Brucella* BacA affect lipid‐a fatty acids. Proceedings of the National Academy of Sciences of the United States of America, 101(14), 5012–5017. 10.1073/pnas.0307137101 15044696 PMC387365

[pld3408-bib-0024] Ferguson, G. P. , Roop, R. M. , & Walker, G. C. (2002). Deficiency of a *Sinorhizobium meliloti bacA* mutant in alfalfa symbiosis correlates with alteration of the cell envelope. Journal of Bacteriology, 184, 5625–5632. 10.1128/JB.184.20.5625-5632.2002 12270820 PMC139620

[pld3408-bib-0025] Finan, T. M. , Hirsch, A. M. , Leigh, J. A. , Johansen, E. , Kuldau, G. A. , Deegan, S. , Walker, G. C. , & Signer, E. R. (1985). Symbiotic mutants of *Rhizobium meliloti* that uncouple plant from bacterial differentiation. Cell, 40, 869–877. 10.1016/0092-8674(85)90346-0 2985267

[pld3408-bib-0026] Gage, D. J. (2004). Infection and invasion of roots by symbiotic, nitrogen‐fixing rhizobia during nodulation of temperate legumes. Microbiology and Molecular Biology Reviews, 68, 280–300. 10.1128/MMBR.68.2.280-300.2004 15187185 PMC419923

[pld3408-bib-0027] Geddes, B. A. , Kearsley, J. V. S. , Huang, J. , Zamani, M. , Muhammed, Z. , Sather, L. , Panchal, A. K. , & Finan, T. M. (2021). Minimal gene set from *Sinorhizobium* (*Ensifer*) *meliloti* pSymA required for efficient symbiosis with *Medicago* . Proceedings of the National Academy of Sciences of the United States of America, 118, e2018015118. 10.1073/pnas.2018015118 33384333 PMC7814474

[pld3408-bib-0028] Glazebrook, J. , Ichige, A. , & Walker, G. C. (1993). A *Rhizobium meliloti* homolog of the *Escherichia coli* peptide‐antibiotic transport protein SbmA is essential for bacteroid development. Genes & Development, 7(8), 1485–1497. 10.1101/gad.7.8.1485 8393417

[pld3408-bib-0029] Grabherr, M. G. , Haas, B. J. , Yassour, M. , Levin, J. Z. , Thompson, D. A. , Amit, I. , Adiconis, X. , Fan, L. , Raychowdhury, R. , Zeng, Q. , Chen, Z. , Mauceli, E. , Hacohen, N. , Gnirke, A. , Rhind, N. , Di Palma, F. , Birren, B. W. , Nusbaum, C. , Lindblad‐Toh, K. , … Regev, A. (2011). Full‐length transcriptome assembly from RNA‐Seq data without a reference genome. Nature Biotechnology, 29(7), 644–652. 10.1038/nbt.1883 PMC3571712 21572440

[pld3408-bib-0030] Guefrachi, I. , Pierre, O. , Timchenko, T. , Alunni, B. , Barrière, Q. , Czernic, P. , Villaécija‐Aguilar, J. A. , Verly, C. , Bourge, M. , Fardoux, J. , Mars, M. , Kondorosi, E. , Giraud, E. , & Mergaert, P. (2015). *Bradyrhizobium* BclA is a peptide transporter required for bacterial differentiation in symbiosis with *Aeschynomene* legumes. Molecular Plant‐Microbe Interactions, 28, 1155–1166. 10.1094/MPMI-04-15-0094-R 26106901

[pld3408-bib-0031] Haag, A. F. , Baloban, M. , Sani, M. , Kerscher, B. , Pierre, O. , Farkas, A. , Longhi, R. , Boncompagni, E. , Hérouart, D. , DallAngelo, S. , Kondorosi, E. , Zanda, M. , Mergaert, P. , & Ferguson, G. P. (2011). Protection of *Sinorhizobium* against host cysteine‐rich antimicrobial peptides is critical for symbiosis. PLoS Biology, 9(10), e1001169. 10.1371/journal.pbio.1001169 21990963 PMC3186793

[pld3408-bib-0032] Haag, A. F. , & Mergaert, P. (2019). Terminal bacteroid differentiation in the *Medicago*–*Rhizobium* interaction–a tug of war between plant and bacteria. The Model Legume Medicago truncatula, 2, 600–616. 10.1002/9781119409144.ch75

[pld3408-bib-0033] Haft, D. H. , Selengut, J. D. , Richter, R. A. , Harkins, D. , Basu, M. K. , & Beck, E. (2012). TIGRFAMs and genome properties in 2013. Nucleic Acids Research, 41(D1), D387–D395. 10.1093/nar/gks1234 23197656 PMC3531188

[pld3408-bib-0034] Honma, M. A. , & Ausubel, F. M. (1987). *Rhizobium meliloti* has three functional copies of the *nodD* symbiotic regulatory gene. Proceedings of the National Academy of Sciences of the United States of America, 84(23), 8558–8562. 10.1073/pnas.84.23.8558 3479806 PMC299584

[pld3408-bib-0035] Horváth, B. , Domonkos, Á. , Kereszt, A. , Szűcs, A. , Ábrahám, E. , Ayaydin, F. , Bóka, K. , Chen, Y. , Chen, R. , Murray, J. D. , Udvardi, M. K. , Kondorosi, É. , & Kaló, P. (2015). Loss of the nodule‐specific cysteine rich peptide, NCR169, abolishes symbiotic nitrogen fixation in the *Medicago truncatula dnf7* mutant. Proceedings of the National Academy of Sciences of the United States of America, 112(49), 15232–15237. 10.1073/pnas.1500777112 26401023 PMC4679056

[pld3408-bib-0036] Huerta‐Cepas, J. , Szklarczyk, D. , Heller, D. , Hernández‐Plaza, A. , Forslund, S. K. , Cook, H. , Mende, D. R. , Letunic, I. , Rattei, T. , Jensen, L. J. , Von Mering, C. , & Bork, P. (2019). EggNOG 5.0: A hierarchical, functionally and phylogenetically annotated orthology resource based on 5090 organisms and 2502 viruses. Nucleic Acids Research, 47, D309–D314. 10.1093/nar/gky1085 30418610 PMC6324079

[pld3408-bib-0037] Ichige, A. , & Walker, G. C. (1997). Genetic analysis of the *Rhizobium meliloti bacA* gene: Functional interchangeability with the *Escherichia coli sbmA* gene and phenotypes of mutants. Journal of Bacteriology, 179, 209–216. 10.1128/jb.179.1.209-216.1997 8982000 PMC178681

[pld3408-bib-0038] Jensen, H. L. (1942). Nitrogen fixation in leguminous plants. I. General characters of root‐nodule bacteria isolated from species of *Medicago* and *Trifolium* in Australia. Proceedings of the Linnean Society of New South Wales, 66, 98–108.

[pld3408-bib-0039] Karaki, L. , Silva, P. D. , Rizk, F. , Chouabe, C. , Chantret, N. , Eyraud, V. , Gressent, F. , Sivignon, C. , Rahioui, I. , Kahn, D. , Brochier‐Armanet, C. , Rahbé, Y. , & Royer, C. (2016). Genome‐wide analysis identifies gain and loss/change of function within the small multigenic insecticidal albumin 1 family of *Medicago truncatula* . BMC Plant Biology, 16, 63. 10.1186/s12870-016-0745-0 26964738 PMC4785745

[pld3408-bib-0040] Kerr, S. C. , Gaiti, F. , Beveridge, C. A. , & Tanurdzic, M. (2017). De novo transcriptome assembly reveals high transcriptional complexity in *Pisum sativum* axillary buds and shows rapid changes in expression of diurnally regulated genes. BMC Genomics, 18, 221. 10.1186/s12864-017-3577-x 28253862 PMC5335751

[pld3408-bib-0041] Kim, M. , Chen, Y. , Xi, J. , Waters, C. , Chen, R. , & Wang, D. (2015). An antimicrobial peptide essential for bacterial survival in the nitrogen‐fixing symbiosis. Proceedings of the National Academy of Sciences of the United States of America, 112(49), 15238–15243. 10.1073/pnas.1500123112 26598690 PMC4679048

[pld3408-bib-0042] Kosslak, R. M. , Bookland, R. , Barkei, J. , Paaren, H. E. , & Appelbaum, E. R. (1987). Induction of *Bradyrhizobium japonicum* common *nod* genes by isoflavones isolated from *Glycine max* . Proceedings of the National Academy of Sciences of the United States of America, 84(21), 7428–7432. 10.1073/pnas.84.21.7428 16593884 PMC299309

[pld3408-bib-0043] Lamouche, F. , Bonadé‐Bottino, N. , Mergaert, P. , & Alunni, B. (2019). Symbiotic efficiency of spherical and elongated bacteroids in the *Aeschynomene*‐*Bradyrhizobium* symbiosis. Frontiers in Plant Science, 10, 377. 10.3389/fpls.2019.00377 31001301 PMC6454206

[pld3408-bib-0044] Leigh, J. A. , Signer, E. R. , & Walker, G. C. (1985). Exopolysaccharide‐deficient mutants of *Rhizobium meliloti* that form ineffective nodules. Proceedings of the National Academy of Sciences of the United States of America, 82(18), 6231 LP–6235. 10.1073/pnas.82.18.6231 3862129 PMC391026

[pld3408-bib-0045] Li, W. , & Godzik, A. (2006). Cd‐hit: A fast program for clustering and comparing large sets of protein or nucleotide sequences. Bioinformatics, 22(13), 1658–1659. 10.1093/bioinformatics/btl158 16731699

[pld3408-bib-0046] Lima, R. M. , Kylarová, S. , Mergaert, P. , & Kondorosi, É. (2020). Unexplored arsenals of legume peptides with potential for their applications in medicine and agriculture. Frontiers in Microbiology, 11, 1307. 10.3389/fmicb.2020.01307 32625188 PMC7314904

[pld3408-bib-0047] Limpens, E. , Moling, S. , Hooiveld, G. , Pereira, P. A. , Bisseling, T. , Becker, J. D. , & Küster, H. (2013). Cell‐and tissue‐specific transcriptome analyses of *Medicago truncatula* root nodules. PLoS ONE, 8(5), e64377. 10.1371/journal.pone.0064377 23734198 PMC3667139

[pld3408-bib-0048] Love, M. , Anders, S. , & Huber, W. (2014). Differential analysis of count data–the DESeq2 package. Genome Biology, 15, 10–1186.

[pld3408-bib-0049] Lukashin, A. V. , & Borodovsky, M. (1998). GeneMark. Hmm: New solutions for gene finding. Nucleic Acids Research, 26(4), 1107–1115. 10.1093/nar/26.4.1107 9461475 PMC147337

[pld3408-bib-0050] Malovichko, Y. V. , Shtark, O. Y. , Vasileva, E. N. , Nizhnikov, A. A. , & Antonets, K. S. (2020). Transcriptomic insights into mechanisms of early seed maturation in the garden pea (*Pisum sativum* L.). Cells, 9(3), 779. 10.3390/cells9030779 PMC7140803 32210065

[pld3408-bib-0051] Marlow, V. L. , Haag, A. F. , Kobayashi, H. , Fletcher, V. , Scocchi, M. , Walker, G. C. , & Ferguson, G. P. (2009). Essential role for the BacA protein in the uptake of a truncated eukaryotic peptide in *Sinorhizobium meliloti* . Journal of Bacteriology, 191(5), 1519–1527. 10.1128/JB.01661-08 19074376 PMC2648212

[pld3408-bib-0052] Maróti, G. , Downie, J. A. , & Kondorosi, É. (2015). Plant cysteine‐rich peptides that inhibit pathogen growth and control rhizobial differentiation in legume nodules. Current Opinion in Plant Biology, 26, 57–63. 10.1016/j.pbi.2015.05.031 26116977

[pld3408-bib-0053] Maróti, G. , Kereszt, A. , Kondorosi, E. , & Mergaert, P. (2011). Natural roles of antimicrobial peptides in microbes, plants and animals. Research in Microbiology, 162(4), 363–374. 10.1016/j.resmic.2011.02.005 21320593

[pld3408-bib-0054] Martin, M. (2011). Cutadapt removes adapter sequences from high‐throughput sequencing reads. EMBnet. Journal, 17, 10–12. 10.14806/ej.17.1.200

[pld3408-bib-0055] Maruya, J. , & Saeki, K. (2010). The *bacA* gene homolog, *mlr7400*, in *Mesorhizobium loti* MAFF303099 is dispensable for symbiosis with *Lotus japonicus* but partially capable of supporting the symbiotic function of *bacA* in *Sinorhizobium meliloti* . Plant and Cell Physiology, 51(9), 1443–1452. 10.1093/pcp/pcq114 20668224

[pld3408-bib-0056] Maxwell, C. A. , Hartwig, U. A. , Joseph, C. M. , & Phillips, D. A. (1989). A chalcone and two related flavonoids released from alfalfa roots induce nod genes of *Rhizobium meliloti* . Plant Physiology, 91(3), 842–847. 10.1104/pp.91.3.842 16667146 PMC1062085

[pld3408-bib-0057] Mergaert, P. , Nikovics, K. , Kelemen, Z. , Maunoury, N. , Vaubert, D. , Kondorosi, A. , & Kondorosi, E. (2003). A novel family in *Medicago truncatula* consisting of more than 300 nodule‐specific genes coding for small, secreted polypeptides with conserved cysteine motifs. Plant Physiology, 132, 161–173. 10.1104/pp.102.018192 12746522 PMC166962

[pld3408-bib-0058] Mergaert, P. , Uchiumi, T. , Alunni, B. , Evanno, G. , Cheron, A. , Catrice, O. , Mausset, A. E. , Barloy‐Hubler, F. , Galibert, F. , Kondorosi, A. , & Kondorosi, E. (2006). Eukaryotic control on bacterial cell cycle and differentiation in the Rhizobium–legume symbiosis. Proceedings of the National Academy of Sciences of the United States of America, 103(13), 5230–5235. 10.1073/pnas.0600912103 16547129 PMC1458823

[pld3408-bib-0059] Mikuláss, K. R. , Nagy, K. , Bogos, B. , Szegletes, Z. , Kovács, E. , Farkas, A. , Váró, G. , Kondorosi, É. , & Kereszt, A. (2016). Antimicrobial nodule‐specific cysteine‐rich peptides disturb the integrity of bacterial outer and inner membranes and cause loss of membrane potential. Annals of Clinical Microbiology and Antimicrobials, 15, 43. 10.1186/s12941-016-0159-8 27465344 PMC4964015

[pld3408-bib-0060] Mistry, J. , Chuguransky, S. , Williams, L. , Qureshi, M. , Salazar, G. A. , Sonnhammer, E. L. L. , Tosatto, S. C. E. , Paladin, L. , Raj, S. , Richardson, L. J. , Finn, R. D. , & Bateman, A. (2021). Pfam: The protein families database in 2021. Nucleic Acids Research, 49(D1), D412–D419. 10.1093/nar/gkaa913 33125078 PMC7779014

[pld3408-bib-0061] Montiel, J. , Downie, J. A. , Farkas, A. , Bihari, P. , Herczeg, R. , Bálint, B. , Mergaert, P. , Kereszt, A. , & Kondorosi, É. (2017). Morphotype of bacteroids in different legumes correlates with the number and type of symbiotic NCR peptides. Proceedings of the National Academy of Sciences of the United States of America, 114(19), 5041–5046. 10.1073/pnas.1704217114 28438996 PMC5441718

[pld3408-bib-0062] Nash, D. T. , & Schulman, H. M. (1976). Leghemoglobins and nitrogenase activity during soybean root nodule development. Canadian Journal of Botany, 54(24), 2790–2797. 10.1139/b76-298

[pld3408-bib-0071] Nicoud, Q. , Barrière, Q. , Busset, N. , Dendene, S. , Travin, D. , Bourge, M. , Le Bars, R. , Boulogne, C. , Lecroël, M. , Jenei, S. , Kereszt, A. , Kondorosi, E. , Biondi, E. G. , Timchenko, T. , Alunni, B. , & Mergaert, P. (2021). *Sinorhizobium meliloti* functions required for resistance to antimicrobial NCR peptides and bacteroid differentiation. mBio, 12, e0089521. https://10.1128.mBio.00895-21 34311575 10.1128/mBio.00895-21 PMC8406287

[pld3408-bib-0063] Oldroyd, G. E. D. (2013). Speak, friend, and enter: Signalling systems that promote beneficial symbiotic associations in plants. Nature Reviews Microbiology, 11(4), 252–263. 10.1038/nrmicro2990 23493145

[pld3408-bib-0064] Ördögh, L. , Vörös, A. , Nagy, I. , Kondorosi, É. , & Kereszt, A. (2014). Symbiotic plant peptides eliminate *Candida albicans* both *in vitro* and in an epithelial infection model and inhibit the proliferation of immortalized human cells. BioMed Research International, 2014, 320796. 10.1155/2014/320796 25243129 PMC4163382

[pld3408-bib-0065] Ott, T. , Dongen, J. T. v. , Gu, C. , Gu¨nther, C. , Krusell, L. , Desbrosses, G. , Vigeolas, H. , Bock, V. , Czechowski, T. , Geigenberger, P. , & Udvardi, M. K. (2005). Symbiotic leghemoglobins are crucial for nitrogen fixation in legume root nodules but not for general plant growth and development. Current Biology, 15(6), 531–535. 10.1016/j.cub.2005.01.042 15797021

[pld3408-bib-0066] Patro, R. , Duggal, G. , Love, M. I. , Irizarry, R. A. , & Kingsford, C. (2017). Salmon provides fast and bias‐aware quantification of transcript expression. Nature Methods, 14, 417–419. 10.1038/nmeth.4197 28263959 PMC5600148

[pld3408-bib-0067] Pecrix, Y. , Staton, S. E. , Sallet, E. , Lelandais‐Brière, C. , Moreau, S. , Carrère, S. , Blein, T. , Jardinaud, M. F. , Latrasse, D. , Zouine, M. , Zahm, M. , Kreplak, J. , Mayjonade, B. , Satgé, C. , Perez, M. , Cauet, S. , Marande, W. , Chantry‐Darmon, C. , Lopez‐Roques, C. , … Gamas, P. (2018). Whole‐genome landscape of Medicago truncatula symbiotic genes. Nature Plants, 4(12), 1017–1025. 10.1038/s41477-018-0286-7 30397259

[pld3408-bib-0068] Petersen, T. N. , Brunak, S. , Von Heijne, G. , & Nielsen, H. (2011). SignalP 4.0: Discriminating signal peptides from transmembrane regions. Nature Methods, 8(10), 785–786. 10.1038/nmeth.1701 21959131

[pld3408-bib-0069] Pueppke, S. G. , Bolanos‐Vásquez, M. C. , Werner, D. , Bec‐Ferté, M.‐P. , Promé, J.‐C. , & Krishnan, H. B. (1998). Release of flavonoids by the soybean cultivars McCall and Peking and their perception as signals by the nitrogen‐fixing symbiont *Sinorhizobium fredii* . Plant Physiology, 117(2), 599–606. 10.1104/pp.117.2.599 9625713 PMC34980

[pld3408-bib-0070] Pueppke, S. G. , & Broughton, W. J. (1999). *Rhizobium* sp. strain NGR234 and *R. fredii* USDA257 share exceptionally broad, nested host ranges. Molecular Plant‐Microbe Interactions, 12(4), 293–318. 10.1094/MPMI.1999.12.4.293 10188270

[pld3408-bib-0072] Rahioui, I. , Eyraud, V. , Karaki, L. , Sasse, F. , Carre‐Pierrat, M. , Qin, A. , Zheng, M. H. , Toepfer, S. , Sivignon, C. , Royer, C. , Da Silva, P. , & Gressent, F. (2014). Host range of the potential biopesticide pea albumin 1b (PA1b) is limited to insects. Toxicon, 89, 67–76. 10.1016/j.toxicon.2014.07.004 25064271

[pld3408-bib-0073] Recourt, K. , Schripsema, J. , Kijne, J. W. , van Brussel, A. A. N. , & Lugtenberg, B. J. J. (1991). Inoculation of *Vicia sativa* subsp. *nigra* roots with *Rhizobium leguminosarum* biovar *viciae* results in release of *nod* gene activating flavanones and chalcones. Plant Molecular Biology, 16(5), 841–852. 10.1007/BF00015076 1859867

[pld3408-bib-0074] Roux, B. , Rodde, N. , Jardinaud, M.‐F. , Timmers, T. , Sauviac, L. , Cottret, L. , Carrère, S. , Sallet, E. , Courcelle, E. , Moreau, S. , Debellé, F. , Capela, D. , De Carvalho‐Niebel, F. , Gouzy, J. , Bruand, C. , & Gamas, P. (2014). An integrated analysis of plant and bacterial gene expression in symbiotic root nodules using laser‐capture microdissection coupled to RNA sequencing. The Plant Journal, 77(6), 817–837. 10.1111/tpj.12442 24483147

[pld3408-bib-0075] Sallet, E. , Roux, B. , Sauviac, L. , Jardinaud, M. F. , Carrere, S. , Faraut, T. , De Carvalho‐Niebel, F. , Gouzy, J. , Gamas, P. , Capela, D. , Bruand, C. , & Schiex, T. (2013). Next‐generation annotation of prokaryotic genomes with EuGene‐P: Application to *Sinorhizobium meliloti* 2011. DNA Research, 20(4), 339–354. 10.1093/dnares/dst014 23599422 PMC3738161

[pld3408-bib-0076] Seppey, M. , Manni, M. , & Zdobnov, E. M. (2019). BUSCO: Assessing genome assembly and annotation completeness. In Gene Prediction (Vol. 1962) (pp. 227–245. 10.1007/978-1-4939-9173-0_14). Springer. 31020564

[pld3408-bib-0077] Sievers, F. , Wilm, A. , Dineen, D. , Gibson, T. J. , Karplus, K. , Li, W. , Lopez, R. , McWilliam, H. , Remmert, M. , Söding, J. , Thompson, J. D. , & Higgins, D. G. (2011). Fast, scalable generation of high‐quality protein multiple sequence alignments using Clustal omega. Molecular Systems Biology, 7(1), 539. 10.1038/msb.2011.75 21988835 PMC3261699

[pld3408-bib-0078] Simsek, S. , Ojanen‐Reuhs, T. , Stephens, S. B. , & Reuhs, B. L. (2007). Strain‐ecotype specificity in Sinorhizobium meliloti‐Medicago truncatula symbiosis is correlated to succinoglycan oligosaccharide structure. Journal of Bacteriology, 189(21), 7733–7740. 10.1128/JB.00739-07 17766412 PMC2168717

[pld3408-bib-0079] Song, L. , & Florea, L. (2015). Rcorrector: Efficient and accurate error correction for Illumina RNA‐seq reads. GigaScience, 4(1), s13742–s13015. 10.1186/s13742-015-0089-y PMC4615873 26500767

[pld3408-bib-0080] Stanke, M. , Schöffmann, O. , Morgenstern, B. , & Waack, S. (2006). Gene prediction in eukaryotes with a generalized hidden Markov model that uses hints from external sources. BMC Bioinformatics, 7, 1–11. 10.1186/1471-2105-7-62 16469098 PMC1409804

[pld3408-bib-0081] Tiricz, H. , Szűcs, A. , Farkas, A. , Pap, B. , Lima, R. M. , Maróti, G. , Kondorosi, É. , & Kereszt, A. (2013). Antimicrobial nodule‐specific cysteine‐rich peptides induce membrane depolarization‐associated changes in the transcriptome of *Sinorhizobium meliloti* . Applied and Environmental Microbiology, 79(21), 6737–6746. 10.1128/AEM.01791-13 23995935 PMC3811505

[pld3408-bib-0014] The UniProt Consortium . (2021). UniProt: The universal protein knowledgebase in 2021. Nucleic Acids Research, 49(D1), D480–D489. 10.1093/nar/gkaa1100 33237286 PMC7778908

[pld3408-bib-0082] Tsukui, T. , Eda, S. , Kaneko, T. , Sato, S. , Okazaki, S. , Kakizaki‐Chiba, K. , Itakura, M. , Mitsui, H. , Yamashita, A. , Terasawa, K. , & Minamisawa, K. (2013). The type III secretion system of *Bradyrhizobium japonicum* USDA122 mediates symbiotic incompatibility with Rj2 soybean plants. Applied and Environmental Microbiology, 79(3), 1048–1051. 10.1128/AEM.03297-12 23204412 PMC3568557

[pld3408-bib-0083] Tsurumaru, H. , Hashimoto, S. , Okizaki, K. , Kanesaki, Y. , Yoshikawa, H. , & Yamakawa, T. (2015). A putative type III secretion system effector encoded by the MA20_12780 gene in *Bradyrhizobium japonicum* Is‐34 causes incompatibility with Rj4 genotype soybeans. Applied and Environmental Microbiology, 81(17), 5812–5819. 10.1128/AEM.00823-15 26092458 PMC4551253

[pld3408-bib-0084] Van de Velde, W. , Zehirov, G. , Szatmari, A. , Van de Velde, W. , Debreczeny, M. , Ishihara, H. , Kevei, Z. , Farkas, A. , Mikulass, K. , Nagy, A. , Tiricz, H. , Satiat‐Jeunemaître, B. , Alunni, B. , Bourge, M. , Kucho, K. I. , Abe, M. , Kereszt, A. , Maroti, G. , Uchiumi, T. , … Mergaert, P. (2010). Plant peptides govern terminal differentiation of bacteria in symbiosis. Science, 327(5969), 1122–1126. 10.1126/science.1184057 20185722

[pld3408-bib-0085] Walker, L. , Lagunas, B. , & Gifford, M. L. (2020). Determinants of host range specificity in legume‐rhizobia Symbiosis. Frontiers in Microbiology, 11, 3028. 10.3389/fmicb.2020.585749 PMC7728800 33329456

[pld3408-bib-0086] Wang, Q. , Liu, J. , Li, H. , Yang, S. , Körmöczi, P. , Kereszt, A. , & Zhu, H. (2018). Nodule‐specific cysteine‐rich peptides negatively regulate nitrogen‐fixing symbiosis in a strain‐specific manner in *Medicago truncatula* . Molecular Plant‐Microbe Interactions, 31, 240–248. 10.1094/MPMI-08-17-0207-R 28990486

[pld3408-bib-0087] Wang, Q. , Yang, S. , Liu, J. , Terecskei, K. , Ábrahám, E. , Gombár, A. , Domonkos, Á. , Szűcs, A. , Körmöczi, P. , Wang, T. , Fodor, L. , Mao, L. , Fei, Z. , Kondorosi, É. , Kaló, P. , Kereszt, A. , & Zhu, H. (2017). Host‐secreted antimicrobial peptide enforces symbiotic selectivity in *Medicago truncatula* . Proceedings of the National Academy of Sciences of the United States of America, 114(26), 6854–6859. 10.1073/pnas.1700715114 28607058 PMC5495241

[pld3408-bib-0088] Wang, T.‐Z. , Liu, M. , Zhao, M.‐G. , Chen, R. , & Zhang, W.‐H. (2015). Identification and characterization of long non‐coding RNAs involved in osmotic and salt stress in *Medicago truncatula* using genome‐wide high‐throughput sequencing. BMC Plant Biology, 15, 131. 10.1186/s12870-015-0530-5 26048392 PMC4457090

[pld3408-bib-0089] Weisberg, A. J. , Kim, G. , Westwood, J. H. , & Jelesko, J. G. (2017). Sequencing and de novo assembly of the *Toxicodendron radicans* (poison ivy) transcriptome. Genes, 8(11), 317. 10.3390/genes8110317 PMC5704230 29125533

[pld3408-bib-0090] Wilson, J. K. (1939). Leguminous Plants and Their Associated Organisms (p. 221). Cornell University Agricultural Experiment Station.

[pld3408-bib-0091] Yang, S. , Tang, F. , Gao, M. , Krishnan, H. B. , & Zhu, H. (2010). R gene‐controlled host specificity in the legume–rhizobia symbiosis. Proceedings of the National Academy of Sciences of the United States of America, 107(43), 18735–18740. 10.1073/pnas.1011957107 20937853 PMC2973005

[pld3408-bib-0092] Yang, S. , Wang, Q. , Fedorova, E. , Liu, J. , Qin, Q. , Zheng, Q. , Price, P. A. , Pan, H. , Wang, D. , Griffitts, J. S. , Bisseling, T. , & Zhu, H. (2017). Microsymbiont discrimination mediated by a host‐secreted peptide in *Medicago truncatula* . Proceedings of the National Academy of Sciences of the United States of America, 114(26), 6848–6853. 10.1073/pnas.1700460114 28607056 PMC5495240

[pld3408-bib-0093] Young, N. D. , Debellé, F. , Oldroyd, G. E. D. , Geurts, R. , Cannon, S. B. , Udvardi, M. K. , Benedito, V. A. , Mayer, K. F. X. , Gouzy, J. , Schoof, H. , Van de Peer, Y. , Proost, S. , Cook, D. R. , Meyers, B. C. , Spannagl, M. , Cheung, F. , De Mita, S. , Krishnakumar, V. , Gundlach, H. , … Roe, B. A. (2011). The *Medicago* genome provides insight into the evolution of rhizobial symbioses. Nature, 480(7378), 520–524. 10.1038/nature10625 22089132 PMC3272368

[pld3408-bib-0094] Zamani, M. , diCenzo, G. C. , Milunovic, B. , & Finan, T. M. (2017). A putative 3‐hydroxyisobutyryl‐CoA hydrolase is required for efficient symbiotic nitrogen fixation in *Sinorhizobium meliloti* and *Sinorhizobium fredii* NGR234. Environmental Microbiology, 19, 218–236. 10.1111/1462-2920.13570 27727485

[pld3408-bib-0095] Zhang, S. , Shi, Y. , Cheng, N. , Du, H. , Fan, W. , & Wang, C. (2015). De novo characterization of fall dormant and nondormant alfalfa (*Medicago sativa* L.) leaf transcriptome and identification of candidate genes related to fall dormancy. PLoS ONE, 10, e0122170. 10.1371/journal.pone.0122170 25799491 PMC4370819

[pld3408-bib-0096] Zhou, P. , Silverstein, K. A. T. , Gao, L. , Walton, J. D. , Nallu, S. , Guhlin, J. , & Young, N. D. (2013). Detecting small plant peptides using SPADA (small peptide alignment discovery application). BMC Bioinformatics, 14, 1–16. 10.1186/1471-2105-14-335 24256031 PMC3924332

