# Supplementary material for: Reference nodule transcriptomes for Melilotus officinalis and Medicago sativa cv. Algonquin
Source: Plant Direct. 2022 Jun 8;6(6):e408. doi: 10.1002/pld3.408 (PMC9219011; doi:10.1002/pld3.408)
Supplement: Supplementary file 8 — Table S1. Shoot dry weights of M. sativa and M. officinalis plants inoculated with Sinorhizobium meliloti strains carrying various bacA constructs. Table S2. Shoot dry weights of M. sativa and M. officinalis plants inoculated with wildtype S. meliloti Rm2011 and used for the transcriptome analyses. Table S3. Number of Illumina paired‐end reads remaining per library following preprocessing. Table S4. Per species summary statistics from the preprocessing of the Illumina reads. Table S5. Summary statistics from annotation of the compressed transcriptome assemblies. Figure S1. Integrity of the RNA samples used for RNA‐seq library preparation. RNA samples were run on a MOPS‐formaldehyde agarose gel and imaged. The bands corresponding to the 28S rRNA, 18S rRNA, and 5.8S rRNA are indicated. The lack of smearing indicates that the purified RNA was of high quality and not degraded. [file PLD3-6-e408-s002.pdf]

Supplementary Material for
Reference nodule transcriptomes for *Melilotus officinalis* and *Medicago sativa* cv. Algonquin

Rui Huang, Wayne A Snedden, George C diCenzo

George diCenzo
Email: george.dicenzo@queensu.ca

This PDF file includes:

Tables S1 to S5
Figure S1
Legends for Datasets S1 to S7

Other supplementary materials for this manuscript include the following:

Datasets S1 to S7

Table S1. Shoot dry weights of *M. sativa* and *M. officinalis* plants inoculated with *S. meliloti* strains carrying various *bacA* constructs.

S. meliloti strain	*bacA* allele	*M. sativa* (mg/plant) *	*M. officinalis* (mg/plant) *
Rm2011	*S. meliloti* Rm2011 *bacA*	52 ± 4 [a]	115 ± 14 [a]
RmP3985	Δ*bacA*	6 ± 3 [b]	5 ± 2 [c]
RmP3903	*S. meliloti* Rm2011 *bacA*	51 ± 8 [a]	131 ± 13 [a]
RmP3905	*S. fredii* NGR234 *bacA*	8 ± 1 [b]	49 ± 9 [b]
RmP3907	*R. leguminosarum* bv. *viciae* 3841 *bacA*	5 ± 3 [b]	123 ± 21 [a]
Uninoculated	N/A	4 ± 2 [b]	4 ± 2 [c]

* Data represent the mean ± the standard deviation of triplicate samples, with each sample consisting of between three and five plants. Different letters represent statistically unique groups ($\alpha < 0.05$) and were determined via one-way ANOVA followed by Tukey's HSD posthoc tests. Statistical analyses were performed separately for each plant species.

Table S2. Shoot dry weights of *M. sativa* and *M. officinalis* plants inoculated with wildtype *S. meliloti* Rm2011 and used for the transcriptome analyses.

Plant	Number of Shoots	Dry Mass per Shoot (mg)
M. sativa replicate 1 *	5	52
M. sativa replicate 2	4	40
M. sativa replicate 3	4	45
M. sativa replicate 4 *	4	42.5
M. sativa replicate 5	4	92.5
M. sativa replicate 6 *	5	46
M. sativa replicate 7	5	56
M. sativa average	4	53
M. officinalis replicate 1	5	64
M. officinalis replicate 2 *	5	63.4
M. officinalis replicate 3 *	5	86
M. officinalis replicate 4	4	90
M. officinalis replicate 5 *	5	76
M. officinalis replicate 6	5	70
M. officinalis replicate 7	5	88
M. officinalis average	5	77

* Asterisks denote replicates used for the transcriptome analyses.

Table S3. Number of Illumina paired-end reads remaining per library following preprocessing.

Library	Paired-end read count
M. sativa 1	39,700,239
M. sativa 4	41,545,950
M. sativa 6	93,460,866
M. officinalis 2	39,420,850
M. officinalis 3	41,842,988
M. officinalis 5	38,069,983

Table S4. Per species summary statistics from the preprocessing of the Illumina reads.

	M. sativa	*M. officinalis*
Raw paired-end reads	202,126,321	138,919,600
Paired-end reads corrected by Rcorrector *	53,303,105 (26.3%)	35,705,018 (25.7%)
Paired-end reads removed by Rcorrector *	20,111,775 (10.0%)	14,629,169 (10.5%)
Paired-end reads retained by Rcorrector *	182,014,546 (90.0%)	124,290,431 (89.5%)
Base pairs trimmed by Cutadapt †	153,232,831 (0.4%)	105,798,758 (0.3%)
Paired-end reads retained by Cutadapt †	182,014,546 (100%)	124,290,431 (100%)
Paired-end reads retained by Trimmomatic ¥	174,707,055 (96.0%)	119,333,821 (96.0%)

* Percentages are given relative to the number of raw paired-end reads.

† Percentages are given relative to the number of paired-end reads retained by Rcorrector or the number of base pairs presented in the paired-end reads retained by Rcorrector.

¥ Percentages are given relative to the number of paired-end reads retained by Cutadapt.

Table S5. Summary statistics from annotation of the compressed transcriptome assemblies.

	M. sativa	*M. officinalis*
Total genes predicted by TransDecoder	73,830	58,786
Genes annotated via bi-directional blast with *Medicago truncatula*	17,446	16,422
Genes annotated using eggNOG-mapper	15,091	10,303
Genes annotated using the HMMs of the PFAM database	843	1,493
Genes annotated using the HMMs of the TIGRFAM database	51	60
Total number of annotated genes	33,431	28,278
Annotation rate (%)	45	48

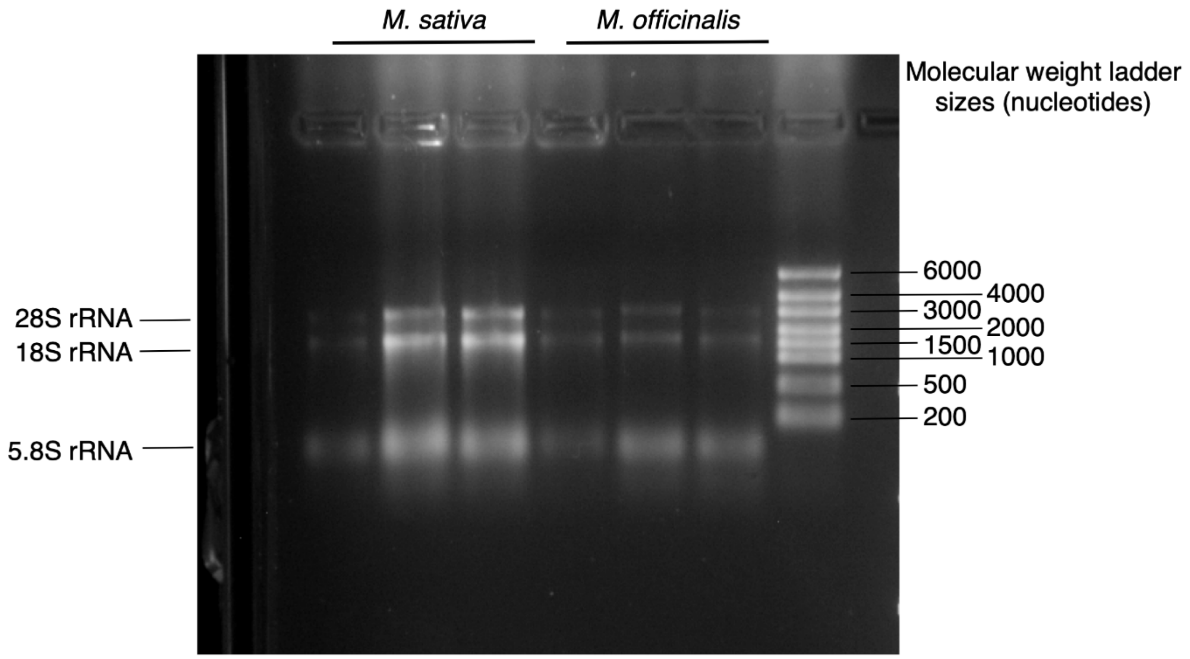

Figure S1. Integrity of the RNA samples used for RNA-seq library preparation. RNA samples were run on a MOPS-formaldehyde agarose gel and imaged. The bands corresponding to the 28S rRNA, 18S rRNA, and 5.8S rRNA are indicated. The lack of smearing indicates that the purified RNA was of high quality and not degraded.

DATASETS

Dataset S1. Annotation of the *Medicato sativa* nodule transcriptome.

Dataset S2. Annotation of the *Melilotus officinalis* nodule transcriptome.

Dataset S3. Summary of the GO slim analysis of the *Medicago sativa* nodule transcriptome assembly.

Dataset S4. Summary of the GO slim analysis of the *Melilotus officinalis* nodule transcriptome assembly.

Dataset S5. NCR peptides predicted from the *Medicago sativa* nodule transcriptome assembly.

Dataset S6. NCR peptides predicted from the *Melilotus officinalis* nodule transcriptome assembly.

Dataset S7. Summary of *M. sativa* and *M. officinalis* NCR peptides amino acid sequences comparsion with *M. truncatula* NCR peptides.
